# Turmeric Powder Counteracts Oxidative Stress and Reduces AFB1 Content in the Liver of Broilers Exposed to the EU Maximum Levels of the Mycotoxin

**DOI:** 10.3390/toxins15120687

**Published:** 2023-12-07

**Authors:** Neenu Amminikutty, Veronica Spalenza, Watanya Jarriyawattanachaikul, Paola Badino, Maria Teresa Capucchio, Elena Colombino, Achille Schiavone, Donato Greco, Vito D’Ascanio, Giuseppina Avantaggiato, Sihem Dabbou, Carlo Nebbia, Flavia Girolami

**Affiliations:** 1 Department of Veterinary Sciences, University of Torino, 10095 Grugliasco, Italy; neenu.amminikutty@unito.it (N.A.); spalenza.veronica@gmail.com (V.S.); w.jarriyawat@gmail.com (W.J.); paola.badino@unito.it (P.B.); mariateresa.capucchio@unito.it (M.T.C.); elena.colombino@edu.unito.it (E.C.); achille.schiavone@unito.it (A.S.); flavia.girolami@unito.it (F.G.); 2 Institute of Sciences of Food Production, Italian National Research Council, 70126 Bari, Italy; donato.greco@ispa.cnr.it (D.G.); vito.dascanio@ispa.cnr.it (V.D.); giuseppina.avantaggiato@ispa.cnr.it (G.A.); 3 Center Agriculture Food Environment (C3A), University of Trento, 38010 San Michele all’Adige, Italy; sihem.dabbou@unitn.it

**Keywords:** aflatoxin B1, turmeric powder, curcumin, oxidative stress, liver, chicken, drug-metabolizing enzymes, drug transporters

## Abstract

The most frequent adverse effects of AFB1 in chicken are low performance, the depression of the immune system, and a reduced quality of both eggs and meat, leading to economic losses. Since oxidative stress plays a major role in AFB1 toxicity, natural products are increasingly being used as an alternative to mineral binders to tackle AFB1 toxicosis in farm animals. In this study, an in vivo trial was performed by exposing broilers for 10 days to AFB1 at dietary concentrations approaching the maximum limits set by the EU (0.02 mg/kg feed) in the presence or absence of turmeric powder (TP) (included in the feed at 400 mg/kg). The aims were to evaluate (i) the effects of AFB1 on lipid peroxidation, antioxidant parameters, histology, and the expression of drug transporters and biotransformation enzymes in the liver; (ii) the hepatic accumulation of AFB1 and its main metabolites (assessed using an in-house-validated HPLC-FLD method); (iii) the possible modulation of the above parameters elicited by TP. Broilers exposed to AFB1 alone displayed a significant increase in lipid peroxidation in the liver, which was completely reverted by the concomitant administration of TP. Although no changes in glutathione levels and antioxidant enzyme activities were detected in any treatment group, AFB1 significantly upregulated and downregulated the mRNA expression of CYP2A6 and Nrf2, respectively. TP counteracted such negative effects and increased the hepatic gene expression of selected antioxidant enzymes (i.e., CAT and SOD2) and drug transporters (i.e., ABCG2), which were further enhanced in combination with AFB1. Moreover, both AFB1 and TP increased the mRNA levels of ABCC2 and ABCG2 in the duodenum. The latter changes might be implicated in the decrease in hepatic AFB1 to undetectable levels (<LOD) in the TP supplemented group. Overall, our findings further support the use of TP as an effective feeding strategy to prevent AFB1-related adverse effects in broilers.

## 1. Introduction

Aflatoxins (AF), secondary metabolites mainly produced by some fungi of the *Aspergillus* genera, are the most studied mycotoxins due to their genotoxic carcinogenic potential and their numerous detrimental effects on important organs/systems, such as the liver, kidney, gut, and the immune system, in both humans and animals [1]. In the last decade, several studies have reported the influence of climate change on fungal proliferation and toxinogenesis, causing an unwanted increase in feed and food contamination [2]. In Southern Europe, elevated temperatures, significant variations in rainfall/drought, and increasing CO_2_ atmospheric concentrations have made the Mediterranean basin a hot zone for the risk of aflatoxin B1 (AFB1) contamination in maize [3]. To protect human and animal health, most countries have set maximum limits for food and feed. In the European Union, they are between 0.005 (dairy ruminants) and 0.02 mg/kg in both complementary and complete feed for farm animals (EU Regulation 574/2011).

The poultry industry is one of the major income-producing sectors in both developing and developed countries; however, it faces a plethora of challenges, with the excessive feed contamination by mycotoxins being one of the most prominent [4]. It is well known that AFB1 impairs the growth performance of broilers, negatively impacting on weight gain, feed intake, feed conversion ratio, and mortality rate [5,6]. Experimental studies performed on chicken have also demonstrated the ability of AFB1 to induce oxidative stress (OS) in various tissues and organs, with a parallel impairment of the antioxidant defense system [7,8]. Such negative effects are related to the peroxidation of membrane phospholipids and oxidative DNA damage with the formation of DNA and protein adducts, especially in the liver. In this respect, the highly reactive AFB1-epoxide (AFBO) resulting from the oxidative AFB1 metabolism is thought to play a key role [9].

Numerous pre- (e.g., genetically modified crops) and post-harvest (i.e., physical/chemical/biological treatments, dietary supplements) strategies have been developed to prevent or mitigate mycotoxin contamination and restore the safety and palatability of feed and food commodities [10]. Nowadays, the incorporation of mycotoxin binders (e.g., clays, aluminosilicates, yeast cell walls) that can complex one or more mycotoxins, reducing their gut absorption, into animal feed is a common practice. However, the major disadvantage of mycotoxin binders is their potential capacity to adsorb vitamins, minerals, amino acids, and other nutrients or feed additives [11]. Hence, there is an increasing interest in the possible use of natural plant-derived products to counteract the negative effects of AFs on animal health through the amelioration or prevention of OS [12]. Should this be realized, phytochemicals would also have the advantage of being biodegradable, cost-effective, eco-friendly, and sustainable.

Curcumin is the major polyphenolic carotenoid extracted from turmeric (*Curcuma longa*), along with other two minor components known as curcuminoids (i.e., demethoxycurcumin and bis-demethoxycurcumin). Their pharmacological properties include antioxidant, anti-inflammatory, chemopreventive, antimicrobial, antiviral, and antifungal effects that have been greatly explored in humans and in several animal species [13,14]. Recently, the turmeric extract (containing at least 90% active substances as the sum of curcumin and curcuminoids) has been authorized in the EU for use as a sensory (i.e., flavoring) feed additive (EU Regulation 2021/551), with a recommended maximum safe concentration of 28 mg/kg in chickens for fattening [15].

The protective role of curcumin and curcuminoids against AFB1 toxicity depends partly on the modulation of the phase I (e.g., CYP1A1, CYP2A6, CYP3A) and phase II (e.g., GSTs) biotransformation enzymes involved in mycotoxin metabolism [16,17,18]. Moreover, they have been proven to interact with the drug transporters (DTs) belonging to the ABC family (e.g., ABCB1, ABCC1, and ACBG2), thus potentially regulating the bioavailability, tissue distribution, and excretion of AF [19,20]. Finally, turmeric bioactive components contribute to counteracting the OS induced by AFB1, increasing the expression/activity of antioxidant enzymes both in vitro and in vivo [7,21,22].

The present study investigated the potential advantages of incorporating turmeric powder (TP) into the diet of broilers exposed to AFB1 at levels approaching the EU maximum limits. The main aims of this study were (i) to assess whether short-term exposure to very low concentrations of AFB1 would elicit hepatic OS, (ii) to test the effects of including TP in the feed at a safe concentration and whether it would improve liver antioxidant status and mitigate the potential pro-oxidant effects of AFB1, and (iii) to examine the effects of TP supplementation on the liver content of AFB1 and its metabolites. An analytical High-Performance Liquid Chromatography with Fluorescence Detection (HPLC-FLD) method was used to determine the AF residues in liver samples at very low levels (ng/Kg), and this method was optimized and validated in-house to ensure high accuracy and sensitivity comparable to that of LC-MS/MS methods.

## 2. Results

### 2.1. Effects of AFB1 and TP on Hepatic Lipid Peroxidation

**Figure 1:**
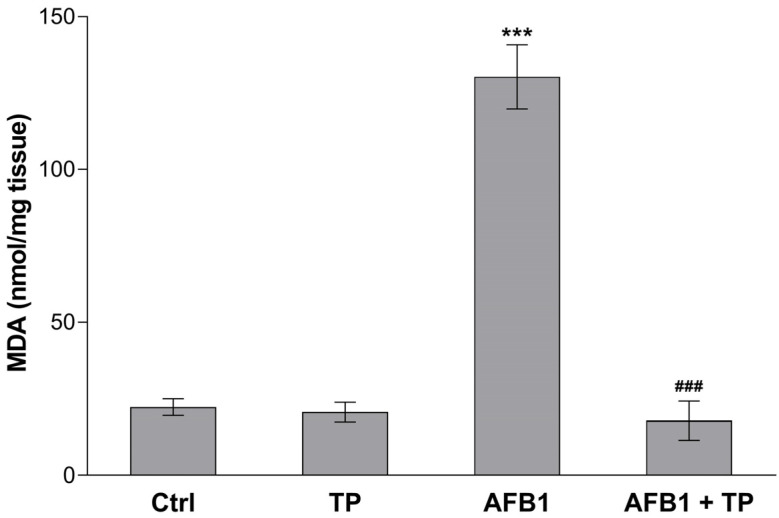
Effects of TP and AFB1 on hepatic lipid peroxidation, measured as MDA levels, in broilers at the end of the treatment period (10 days). Ctrl, control group; TP, turmeric powder group; AFB1, Aflatoxin B1 group; AFB1 + TP, Aflatoxin B1 plus turmeric powder group. Data are expressed as mean ± SEM, *n* = 8 (*** *p* < 0.001 vs. Ctrl; ### *p* < 0.001 vs. AFB1).

The lipid peroxidation of the liver tissues was assessed through the TBARS assay. At the end of the treatment (10 days), malondialdehyde (MDA) levels were significantly augmented by approximately 6-fold in broilers exposed to AFB1 compared to the control group (*** *p* < 0.001 vs. Ctrl). TP alone did not affect the MDA amount in the liver; however, in the co-treatment group (AFB1 + TP), it significantly counteracted the effect of the mycotoxin, decreasing the altered levels to basal values (### *p* < 0.001 vs. AFB1) (Figure 1).

### 2.2. Effects of AFB1 and TP on the Histological Morphology of the Liver Tissues

In the liver tissues of broilers from all experimental groups, no signs of vacuolar degeneration were detected. The only finding was a multifocal lymphoplasmacytic hepatitis, mainly periportal, associated with a multifocal lymphoid hyperplasia. The severity of alterations, scored using a semiquantitative system (range 0–3), varied from absent to mild, and they were accidentally distributed among the experimental groups, without any significant differences on average between the treated and control broilers (Table 1).

### 2.3. Effects of AFB1 and TP on Hepatic GSH Content and GST Catalytic Activities

Since conjugation with GSH catalyzed by the GST family enzymes is the main detoxifying pathway of the AFB1-epoxide, GSH content and the enzymatic activity of total GST, GST α, and GST µ classes were assessed in the liver samples derived from the broilers. Table 2 depicts the effects of the different treatments on GSH levels and GST activities. None of the investigated paraments were affected in a statistically significant way by the exposure to AFB1 and TP, either alone or in combination.

### 2.4. Effects of AFB1 and TP on the Gene Expression of Biotransformation Enzymes, Antioxidant Enzymes, and Drug Transporters (DTs)

**Figure 2:**
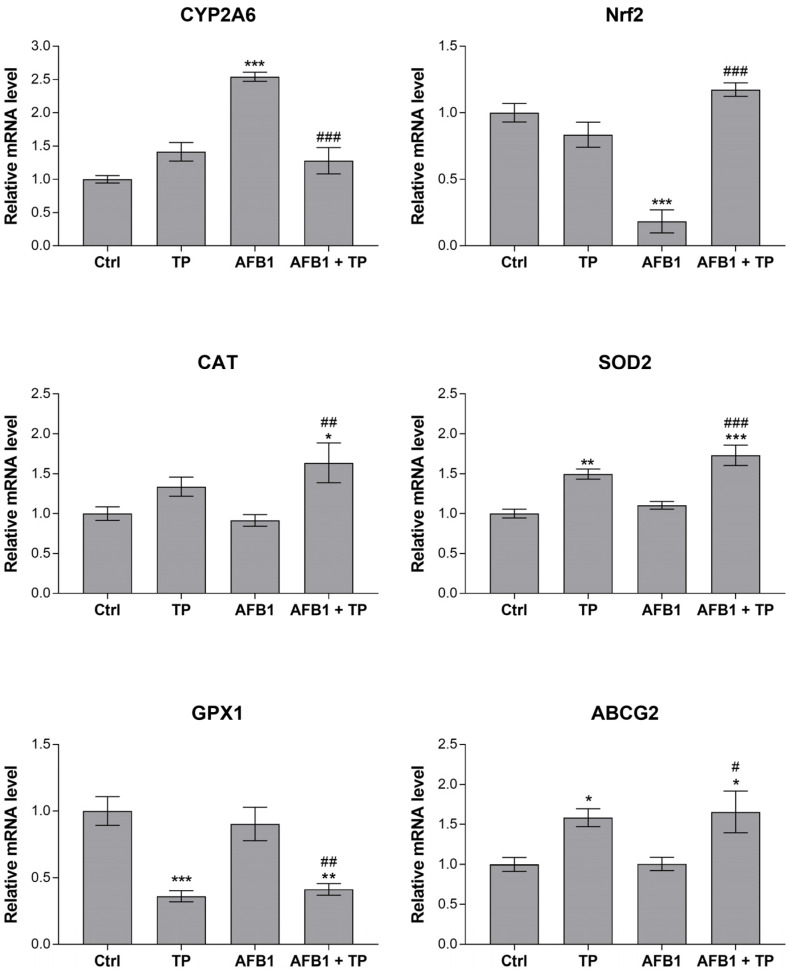
Effects of TP and AFB1 on the mRNA expression of selected genes in the liver of broilers at the end of the treatment period (10 days), measured using qRT-PCR. Ctrl, control group; TP, turmeric powder group; AFB1, Aflatoxin B1 group; AFB1 + TP, Aflatoxin B1 plus turmeric powder group. Data are expressed as mean ± SEM, *n* = 8 (* *p* < 0.5, ** *p* < 0.01, *** *p* < 0.001 vs. Ctrl; # *p* < 0.05, ## *p* < 0.01, ### *p* < 0.001 vs. AFB1).

**Figure 3:**
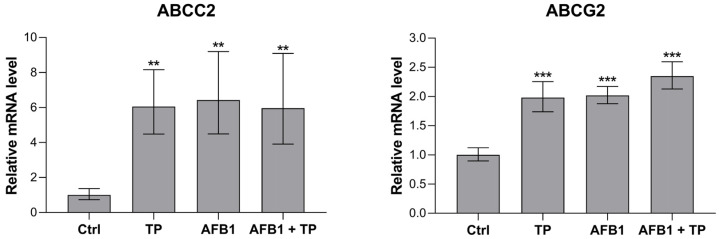
Effects of TP and AFB1 on the mRNA expression of selected genes in the duodenum of broilers at the end of the treatment period (10 days), measured by qRT-PCR. Ctrl, control group; TP, turmeric powder group; AFB1, Aflatoxin B1 group; AFB1 + TP, Aflatoxin B1 plus turmeric powder group. Data are expressed as mean ± SEM, *n* = 8 (** *p* < 0.01, *** *p* < 0.001 vs. Ctrl).

The mRNA expression analysis on the liver samples was performed on a panel of genes, including the enzymes involved in AFB1 metabolism (i.e., CYP1A1, CYP1A2, CYP2A6, CYP2H1, CYP3A4, EPHX1, EPHX2, all isoforms of GSTA, and GSTM2) and in the antioxidant defense (i.e., Nrf2, CAT, GPX1, SOD1, and SOD 2), as well as the DT interacting with curcumin and curcuminoids and AFB1 (i.e., ABCB1, ABCC2, ABCG2). The gene expression study on duodenum samples was performed only on the above-mentioned DT. The quantitative real-time PCR (qRT-PCR) results showed that all the investigated genes were detectable in all the samples. The modulated ones are illustrated in Figure 2 and Figure 3 for the liver and duodenum, respectively.

Among the phase I biotransformation enzymes, CYP2A6 was significantly upregulated by AFB1 by approximately 2.5-fold (*p* < 0.001) compared to the controls. In the broilers of the co-treatment group (AFB1 + TP), TP was able to completely revert the mycotoxin effect (*p* < 0.001). Conversely, AFB1 downregulated Nrf2 by more than 5-fold (*p* < 0.001), and again, such an effect was significantly counteracted by TP (*p* < 0.001), which increased the mRNA levels of Nrf2 to control values in the co-treated broilers. With respect to the antioxidant enzymes, AFB1 did not modulate the expression of any investigated genes, while dietary exposure to TP alone slightly (1.5-fold) but significantly increased the mRNA content of SOD2 compared to the controls (*p* < 0.01). An equivalent augmentation occurred for both SOD2 and CAT in the co-treatment group compared to both the control (*p* < 0.001 and *p* < 0.05) and AFB1-treated broilers (*p* < 0.001 and *p* < 0.01). By contrast, TP reduced the mRNA levels of GPX1 by almost 3-fold in the absence or presence of the mycotoxin (*p* < 0.001 and *p* < 0.01). A similar trend, although less pronounced (1.6-fold), was also observed for EPHX1 (*p* < 0.001 and *p* < 0.05). Finally, the only modulated liver transporter was ABCG2, which was upregulated by approximately 1.6-fold in both the TP and AFB1 + TP groups compared to both the control and mycotoxin-treated broilers (*p* < 0.05).

In intestine samples, the only modulated DTs were ABCC2 and ABCG2, which were significantly upregulated at the mRNA level in all treatment groups compared to the controls. ABCC2 and ABCG2 expression were increased by at least 6- and 2-fold, respectively, with no differences being observed among the groups (i.e., TP, AFB1, or AFB1 + TP).

### 2.5. Effects of TP on the Hepatic Content of AFB1

Very limited amounts of AFB1 were found in liver samples from the control group, with levels less than the LOD (8 ng/kg) in 3 out of 5 specimens (Table 3). In the AF-exposed broilers, the AFB1 concentration was in the 36–81 ng/kg range (mean content ± SEM of 45.9 ± 8.5 ng/kg, *n* = 5), while all samples from the AFB1 + TP group showed AFB1 levels lower than the LOD. No other AF (AFB2, AFG1, and AFG2) or AF metabolites (AFM1, AFM2, AFQ1, and aflatoxicol, AFL) were detected at levels >LOD (Table S3) in livers from all groups.

## 3. Discussion

Moderately/highly AFB1-contaminated diets (range 0.1–5 mg/kg) can impair the functionality of different organs such as the liver and kidney, alter the immunocompetence, and affect both growth performance and egg production [9,23]. Conversely, to the best of our knowledge, few studies have investigated the consequences of administering AFB1 at low dietary concentrations in poultry, and especially in chicken. As liver is the main target organ of AFB1 toxicity and it plays a key role in the biotransformation processes, our study was focused on the hepatic effects caused by the exposure to the mycotoxin and on the ability of TP to tackle such effects.

The results of our in vivo trial showed that broilers exposed to an AFB1-contaminated diet at levels approaching the EU maximum limits (0.02 mg/kg) already display OS in the liver after 10 days. Indeed, the increased lipid peroxidation of hepatic cells (measured as MDA levels) in the AFB1-treated group demonstrated the pro-oxidant effect of the mycotoxin. Moreover, TP dietary supplementation effectively restored the values to control levels, confirming its antioxidant capacity. We previously described a similar scenario in the kidney of the same treated broilers, which also displayed the impairment of blood antioxidant capacity by AFB1 and its counteraction by TP [7]. Our results are in alignment with other studies reporting a significant increase in lipid peroxidation in liver from broilers exposed to AFB1, although at higher concentrations (0.2–5 mg/kg) and for longer periods (21–42 days) [24,25,26,27]. Likewise, the dietary addition of TP at a similar inclusion rate to our study (450 mg/kg feed) was able to alleviate such an effect [28,29].

In most cases, the AFB1-induced OS is coupled with histological alterations like congestion, slight to moderate vacuolar degeneration, and the proliferation of bile ducts [26,30,31]. By contrast, we did not detect any microscopical changes in the liver of the AFB1-treated broilers. Once more, it is worth noting that, in our study, the contaminated feed contained a much lower dose of the mycotoxin (0.02 mg/kg vs. 0.1–0.6 mg/kg), and it was administered for a shorter period (10 days vs. 21–42 days). The continuous production of reactive oxygen species combined with the impairment of the antioxidant defense system mediated by AFB1 (through its active metabolites) is considered a first step in the potential sequence of events leading to cellular toxicity [32]. Thus, it is conceivable that even low doses able to generate OS may result in the onset of cellular lesions at a later stage. To the best of our knowledge, the lowest concentration of AFB1 able to induce mild histological alterations in broiler liver was 0.05 mg/kg diet for an exposure time of 28 days [33].

The major AFB1 detoxification pathway is the GSH conjugation of AFBO, which is mediated by certain GST enzyme families (mainly GST α and GST µ) [34]. No significant changes in the levels of GSH or in the activities of GSTs were found in our experimental animals. A decrease in liver GSH content of 40–50% has been reported in broilers dietarily exposed to AFB1 at concentrations between 0.5 and 5 mg/kg for at least 21 days [22,26,35]. Interestingly, such an effect was also partially or totally restored by co-treatment with TP (300 mg/kg) [36,37]. The outcomes associated with GSH levels at lower dosages of the mycotoxin are controversial: Zhang et al. (2016) observed a significant 30% reduction in the tripeptide after exposing animals to 0.1 mg/kg of AFB1 for 14 days [27]; on the other hand, Balogh et al. (2019) did not detect any significant changes in GSH amount at any time point (days 1–2–3–7–14 of treatment) with 0.15 mg/kg AFB1, similar to our results [38]. Available data about the catalytic activities of the GST families in broiler liver upon AFB1 treatment are limited, and the results are quite divergent, making it difficult to clearly define the impact of AFB1 on such parameters. A significant decrease in total GST activity by almost 40% has been demonstrated following exposure to a highly contaminated diet (5 mg/kg) for 28 days [28]. A similar treatment in terms of duration but at a lower concentration of AFB1 (0.1 mg/kg) did not cause any alterations in GST enzymes, although it was able to induce OS (i.e., increased hepatic MDA levels) [27]. However, a reduction of approximately 30% was reported in broilers treated with an even lower concentration of AFB1 (0.06 mg/kg) for 21 days. Curiously, such a reduction was not associated with an increased MDA content and was less remarkable (10%) after 42 days of treatment [39]. Therefore, even if the comparison with our data is methodologically limited since all the other studies assayed only the total GST activity with unspecified substrates, the lack of changes in the GST families at the catalytic level in our broilers is in line with the complex scenario of pro-oxidant and antioxidant balance.

The expression modulation of genes involved in AFB1 metabolism and in the antioxidant system has already been reported in broiler liver upon exposure to the mycotoxin. Most of the relevant studies have investigated the phase I enzymes responsible for AFB1 bioactivation, namely CYP1A1, CYP1A2, CYP2A6, CYP2H1, and CYP3A4. Although there is evidence of their involvement in the generation of AFBO in poultry, it has been demonstrated that the key role in chicken liver is played by CYP2A6 [40]. Interestingly, in our study, CYP2A6 was the only CYP enzyme significantly induced by AFB1, and such an effect was completely reverted by TP. As far as we know, only a couple of studies have investigated the modulation of CYP2A6 expression by AFB1 (5 mg/kg for 28 days) in broiler liver; in accordance with our data, these studies reported a comparable upregulation of the gene and its counteraction by TP (450 mg/kg) [29,41]. Most researchers have investigated and described the positive gene modulation by AFB1 (0.1–5 mg/kg for at least 21 days) of the other phase I enzymes, especially CYP1A1, CYP1A2, and CYP3A4 [18,27,29,42], which, in our trial, did not significantly change upon treatments. Taking into account the already mentioned leading role of CYP2A6, it might be that, at low concentrations, AFB1 is primarily responsible for the upregulation of such gene, while supplying a higher amount of the mycotoxin for a longer period induces a more robust and generalized modulation of the other bioactivating enzymes.

As far as the genes involved in the antioxidant system are concerned, the main finding in our AFB1-treated broilers is the significant reduction in the hepatic expression of Nrf2, which is a key regulator of cellular resistance to oxidants [43]. Such an effect has already been described in the liver of broilers exposed to 0.5–5 mg/kg AFB1 [22,26,28,36]. Moreover, despite the fact that, in those reports, TP alone did not modulate the mRNA levels of Nrf2, it was able to counteract the pro-oxidant effect of the mycotoxin, in accordance with our results [22,28,36]. The herein-reported AFB1-mediated inhibition of Nrf2 transcription was not matched by a downregulation of the antioxidant enzymes (i.e., CAT, SOD, and GPX). Likewise, a lack of modulation by AFB1 has already been reported for such genes in broiler liver, even at higher exposure levels (0.075–1 mg/kg) [18,44]. In those studies, natural antioxidants such as TP or lipoic acid induced the expression of the antioxidant enzymes, especially in animals fed the combined diet, suggesting their ability to improve overall antioxidant protection in birds fed AFB1. In our study, a similar scenario occurred for CAT and SOD2, while a reverse effect was detected for GPX1, confirming the complex modulation of the antioxidant system by both pro- and antioxidant molecules.

DTs, including ABCB1, ABCC2, and ABCG2, play a key role in the disposition of xenobiotics. In the intestine, they are able to trap and extrude chemicals, hence limiting their oral bioavailability, while in liver, they facilitate their excretion through the biliary and, ultimately, the urinary routes. Little is known about the in vivo ability of mycotoxins and/or TP or curcumin/curcuminoids in relation to the modulation of DT expression in avian species, and the available information is limited to ABCB1 (also referred to as P-gp). An upregulation of jejunal ABCB1 occurred in broiler chicks repeatedly exposed to fumonisins at dietary concentrations approaching the EU guidance levels [45]. Regarding AFB1, exposure to 5 mg/kg feed of the mycotoxin for 28 days slightly decreased the mRNA and protein expression of ABCB1 in the enterocytes, as well as severe histological damage [20]; interestingly, dietary supplementation with TP (range 150–450 mg/kg) reverted both effects but failed to alter ABCB1 expression when administered alone. In line with such findings, pure curcumin was able to upregulate the same transporter in the jejunum of ducklings only at a very high concentration (800 mg/kg) for 21 days [46]. Thus, the lack of modulation of ABCB1 expression in both the intestine and liver of our experimental broilers is consistent with the few in vivo studies performed so far. To the best of our knowledge, this is the first study to investigate ABCC2 and ABCG2 gene expression modulation using AFB1 or TP in avian species. The upregulation of both transporters by AFB1 or curcumin has been described only in vitro on human immortalized cell lines of different tissue origin (i.e., JEG3, MCF-7) [47,48]. Herein, we report an increased expression of ABCG2 in liver and even more in the intestine, driven mainly by TP but also by AFB1 itself, suggesting an altered absorption/excretion of the mycotoxin in the treated broiler chicks (see below for further discussion). On the contrary, no information is available about the role of ABCC2 in the kinetics of AFB1; thus, it is difficult to draw any conclusions about the significance of the upregulation of such efflux pumps observed at the enteric level in our treated broilers.

In our study, the dietary inclusion of very low levels of AFB1 (0.02 mg/kg), only for a short period (10 days), resulted in measurable AFB1 liver concentrations in the 36–81 ng/kg range. Our results are difficult to compare with data from the literature since, in the few reports in which similar toxin concentrations (0.050 to 0.120 mg/kg diet) were used, the exposure duration was much longer (28 to 64 days) [33,49,50,51]. In such studies, AFB1 hepatic levels ranging from <LOQ (40 ng/kg) to 400 ng/kg were found. Taken together, the above data are in line with the good bioavailability of the toxin and the key role played by the liver as the main target organ of AFB1 even following exposure to low toxin amounts [52].

Unexpectedly, administering TP (400 mg/kg feed) to the AFB1-treated broilers resulted in the lack of detectable hepatic levels of AFB1 (<LOD). A possible explanation for this might lie, at least theoretically, in the enhancement of enzymes devoted to AFB1 biotransformation in the liver, resulting in an increase in the amount of the generated metabolites at the expense of the parent compound. However, neither AFM1/AFM2 nor AFL could be recovered even in livers from broilers receiving AFB1 alone. In this respect, our data are in line with those of Ochieng et al. (2023), who did not find AFM1/AFM2 (the only checked metabolites) in livers from broilers administered with higher doses (0.060 or 0.120 mg AFB1/kg feed) for a longer time (35 days) [51]. In partial contrast to our results, Micco et al. (1988) found hepatic undetectable amounts of AFM1, along with 1200 ng/kg AFL, in broilers dietarily treated with 0.060 mg AFB1/kg for 36 days [49]. The different schedule (higher AFB1 doses and a longer treatment period) may explain the mentioned discrepancy. Regarding the potential effects of TP on AFB1 metabolic fate, in our study, neither TP alone nor in combination with AFB1 caused an increase in CYP2A6 or CYP1A1 gene expression, which are mostly implicated in the generation of the oxidated AFB1 metabolites in chicken liver [27,40,52]. More to the point, although we did not examine the effects of the treatments on the expression of the aldo-keto reductase (AKR) performing the reduction of AFB1 to AFL, it should be noted that curcumin has been shown to be one of the most active natural compounds (IC50 80 µM) in AKR inhibition in chicken liver cytosols [16].

The lack of detectable hepatic AFB1 amounts we noticed in the AFB1 + TP treated broilers might also be due to a reduced enteric uptake and/or an enhanced hepatic excretion of the toxin. In the only available in vivo study, the oral bioavailability of AFB1 increased by about two-fold in BCRP/ABCG2 null mice compared to their wild-type counterparts, suggesting an active role for this transporter in the enteric absorption of the toxin [53]. There is also evidence that both AFB1 and AFM1 may be substrates of the bovine BCRP/ABCG2 expressed in MDCKII cells [54]. Of note, the abovementioned fumonisin-mediated upregulation of enteric ABCB1 reported by Antonissen (2017) was linked to a decrease in the oral bioavailability of enrofloxacin [45], a known ABCB1 substrate widely used as an antimicrobial in avian species [55]. A similar decrease in enrofloxacin oral absorption was documented in broiler chickens exposed to relatively low dietary AFB1 concentrations for 42 days [56]. Scant information is available on the kinetic interactions between TP and AFB1 in chickens. According to Cui et al. (2017), curcumin supplementation (300 mg/kg) to AFB1 (5 mg/kg diet)-exposed broilers (28 days of treatment) prolonged the hepatic clearance time of AFB1 (from 11 to 18 days) but slightly shortened that of AFM1 (from 11 to 10 days); no explanations were provided by the authors to justify their findings [57]. In our study, the increased expression of ABCC2 and ABCG2 in the intestine samples (TP, AFB1, and AFB1 + TP groups) and of ABCG2 in the liver samples (TP and AFB1 + TP groups only) points to the interaction among the treatments, with DT regulating the overall kinetics of AFB1 in broilers.

## 4. Conclusions

The short-term dietary administration of AFB1 concentrations around the EU regulatory limits to broiler chicks did not yield significant effects on health status, zootechnical performances, or histological damage. However, as detected by TBARS, it caused marked liver OS, likely the result of the increase in the AFB1 bioactivating enzymes (mainly CYP2A6) and the decrease in the Nrf2 antioxidant response signaling pathway. Dietary supplementation with safer concentrations of TP, according to EFSA [15], reverted the OS and restored both CYP2A6 and Nrf2 gene expression to levels similar to the unexposed individuals. Measurable residues of AFB1 (36–81 ng/kg) but not of its metabolites were found in liver samples from AFB1-treated broilers. On the contrary, AFB1 was undetectable in AFB1 + TP-treated birds, lending support to the hypothesis that the enhanced expression of the DT ABCC2 (intestine) and ABCG2 (intestine and liver) found in both the AFB1 and AFB1 + TP groups could have affected both the absorption and the excretion of the mycotoxin. Further research with increasing dosages of the mycotoxin and a longer exposure time is needed to confirm these results.

## 5. Materials and Methods

### 5.1. Experimental Design and Sample Collection

The in vivo trial was approved by the Institutional Animal Care and Ethic Committee of the University of Turin (Approval number = 319508/2017-PR) and conducted as previously described [7]. Briefly, a total of 32 male 18-day-old broiler chickens (ROSS 308) weighing 751.88 ± 46.28 g were housed in cages in accordance with Directive 2007/43/EC and fed a standard basal diet. After a 4-day acclimation period, they were randomly divided into four experimental groups, each consisting of 8 animals: Ctrl group (basal diet, BD); AFB1 group (BD + 0.02 mg/kg feed AFB1); TP group (BD + 400 mg/kg feed TP); and AFB1 + TP group (BD + 0.02 mg/kg feed AFB1 + 400 mg/kg feed TP). The artificially contaminated feed containing AFB1 was prepared by spiking a blank feed with a fungal culture extract. The amounts of AFB1 and AFB2 in the BD used for the Ctrl group were 5.0 ± 1.3 µg/kg and 0.9 ± 0.1 µg/kg, respectively (mean ± SD, *n* = 3) [7]. TP was added to the basal diet in the form of food-grade turmeric powder (extracted from *Curcuma longa*) (Biorama, Rogeno—LC, Italy) at a concentration of 400 mg/kg feed according to previous studies performed in broilers [18,20]. The content of curcuminoids (the active substances, as the sum of curcumin, desmethoxycurcumin, and bis-desmethoxycurcumin) declared by the producer was equal to 2.5% (85/10/5). The calculated final feed concentration of the turmeric bioactive components was 10 mg/kg, i.e., below the maximum safe concentration suggested by EFSA in chickens for fattening (28 mg/kg) [15]. More details about experimental feed preparation have already been reported by Damiano et al. (2022) [7]. After 10 days of treatment (from 23 to 32 days of age), broilers were sacrificed by an overdose of sodium pentobarbital, and liver and duodenum from each bird were collected and divided into aliquots pending subsequent analyses. Tissue specimens for the lipid peroxidation and enzymatic activities assays, GSH content determination, and chemical analysis were immediately frozen in liquid nitrogen, while samples for the gene expression analysis were placed in RNAlater^®^ stabilization solution (Sigma-Aldrich, Milan, Italy) for 24 h. All samples were then transferred at −80 °C until they were processed. An aliquot of liver from each bird was also fixed in a 10% buffered formalin solution for the histological investigation. During the trial, animal health status was monitored daily; no signs of disease or mortality were reported in all groups. The different dietary treatments did not influence the average daily feed intake, which ranged between 131.7 g/day/bird and 145.1 g/day/bird in all groups.

### 5.2. Thiobarbituric Reactive Substances (TBARS) Assay

Lipid peroxidation in the liver samples was measured using a modified version of the TBARS assay, described by Espin et al. (2017) [58]. An aliquot of liver sample from each broiler was disrupted and homogenized using the Tissue Lyzer LT (Qiagen, Hilden, Germany) for 5 min at 50 Hz in 200 µL of NaCl (0.9% *w*/*v*), 200 µL of TCA (10% *w*/*v*), and 4 µL of butylated hydroxytoluene (BHT) (2% *w*/*v*). The homogenates were centrifuged at 13,000× *g* for 15 min at 4 °C; the supernatants were collected, transferred to precooled tubes, and maintained at 4 °C until analysis to prevent oxidation effects. The 2-thiobarbituric acid (TBA) solution was composed by mixing 15% trichloroacetic acid (*w*/*v*) in glacial acetic acid, 0.38% TBA (*w*/*v*), 0.25N hydrochloric acid, and milliQ-water to the desired final volume. All samples, blanks, and/or MDA standard solutions were vortexed for 1 min after being mixed with 400 µL of TBA solution. MDA-TBA adducts were formed by immersing the samples in a 95° C water bath for 60 min. After 10 min of cooling in a cold water bath to stop the reaction, the samples were centrifuged at 13,000× *g* for 15 min, and the absorbance was measured at 532 nm. All samples, including the blank and the MDA standard curve, were run in triplicate. The results were expressed as nmol of MDA per mg of tissue.

### 5.3. Histological Investigation

The formalin-fixed tissues were routinely embedded in paraffin wax blocks, sectioned at 5 μm thickness, mounted on glass slides, and stained with Hematoxylin & Eosin (H&E). Histopathological alterations such as hepatocyte degeneration and changes in the amount of lymphoplasmacytic inflammatory infiltrates were evaluated using a semiquantitative scoring system with the following descriptors: absent (score = 0), mild (score = 1), moderate (score = 2), and severe (score = 3).

### 5.4. Total GSH Content Determination and Enzymatic Activity Assays

An aliquot of liver sample from each broiler was homogenized, and hepatic subfractions were isolated by differential ultracentrifugation [59]. Protein content was determined using bovine serum albumin as a reference standard [60]. The total GSH content was assayed in cytosolic fractions using dithio-bis-nitrobenzoic acid (DTNB) on TCA-deproteinized samples as previously described [61]. The results were expressed as μmol of GSH per g of liver. The activities of cytosolic total GST and of GST µ were assayed in a final volume of 1.5 mL by the continuous monitoring (at λ = 340 nm) of the GSH conjugates of 1 mM 1-chloro, 2,4-dinitrobenzene (CDNB) and 1 mM 3,4-dichloronitrobenzene (DCNB) [62], respectively, as detailed in Gusson et al. (2006) [63]. The activity of the α class GST was determined spectrophotometrically at 340 nm with tert-butyl hydroperoxide 1.5 mM as the substrate in a final volume of 1 mL, according to Reddy et al. (1981) [64]. All enzyme activities were measured under Vmax conditions and were linear with respect to time and protein concentrations. The results were all expressed as nmol/min per mg of protein.

### 5.5. RNA Extraction and qRT-PCR

Total RNA from the liver and duodenum samples was extracted using the Maxwell RSC simplyRNA Kit according to the manufacturer’s protocol and quantified through uusing the NanoDrop ND-2000 UV-Vis spectrophotometer (Thermo Fisher Scientific, Waltham, MA, USA). The ratio of the optical densities measured at 260 and 280 nm were >1.9 for all RNA samples. RNA integrity was assessed using an automated electrophoresis station (Experion Instrument, Bio-Rad, Hercules, CA, USA). All the samples had an RNA Integrity Number (RIN) > 7. Next, 1 μg of total RNA from each sample was retrotranscribed into complementary DNA (cDNA) using the iScriptTM cDNA Synthesis Kit (BIORAD, Hercules, CA, USA) following the manufacturer’s instructions in a final volume of 20 μL using the GeneAmp PCR System 9700 (Perkin Elmer, Waltham, MA, USA). The primers were designed on the *Gallus gallus* GeneBank database, and Ensembl mRNA sequences using Primer3 software (version 3.0, Applied Biosystems, Foster City, CA, USA). Oligonucleotides were designed to cross the exon/exon boundaries to reduce the amplification of contaminant genomic DNA and were analyzed for the formation of hairpin structures and dimers using the NetPrimer tool (available at http://www.premierbiosoft.com/netprimer/index.html, access date: 15 March 2023). The sequence identity was then confirmed using the blastn function in the bioinformatics tool BLAST (Basic local alignment search tool; https://blast.ncbi.nlm.nih.gov/Blast.cgi, access date: 15 March 2023). Primer information (sequences, gene accession numbers, primer melting temperatures, and amplicon sizes) of target and candidate internal control genes (ICGs) are summarized in Table S1. To identify the most stable couple of ICGs for each tissue, mRNA levels of a set of candidate genes (GAPDH, GUSB, HMBS, HPRT, PGK2, RPL13, RPL19, RPS7, SDHA, TFRC, VIM, and YWHAZ9) were measured in the chicken liver and duodenum samples and analyzed using NormFinder version 0.953 (Andersen et al., 2004) according to the developer’s recommendations [65]. PGK2/RPS7 and GUSB/HPRT were identified as the best combinations of ICGs for the liver and duodenum, respectively. Real-time PCR was performed using the ABI 7500 Real-time PCR System (Applied Biosystems) using 96-well optical plates under the following conditions: 30 s at 95 °C for polymerase activation, 40 cycles of 15 s at 95 °C, and 60 s at 60 °C. Each qRT-PCR reaction was run in triplicate to reduce the intra-assay variability, and a no-template control was included through using water instead of cDNA. Dissociation curves for each gene are reported in Figure S1. The modulation of gene expression was calculated with the 2^−ΔΔCt^ method [66], using the geometric mean of the two best ICGs as a reference [67], and was expressed as relative mRNA level.

### 5.6. Chemical Analysis

#### 5.6.1. Chemicals and Reagents

Unless otherwise stated, all chemicals were of analytical grade. Acetonitrile and methanol were supplied by Carlo Erba Reagents srl (Milan, Italy). Water was of Milli-Q^®^ quality (Millipore, Bedford, MA, USA). β-glucuronidase/aryl sulfatase enzyme solution (30/60 U/mL) was purchased from Merck KGaA (Darmstadt, Germany). AFQ1 was provided by Cfm Oskar Tropitzsch GmbH (Marktredwitz, Germany). The other AF standards (AFB1, AFB2, AFG1, AFG2, AFM1, AFM2, AFL) were provided by Fermentek Ltd. (Jerusalem, Israel). All AF stock solutions (1 µg/mL) were prepared in acetonitrile and stored in the dark at 4 °C. AflaTest™ WB immunoaffinity columns (IMA) were provided by VICAM (Waters Corporation, Milford, MA, USA).

#### 5.6.2. Liver Sample Preparation

The liver samples were thawed, weighed, and homogenized using a pestle to break down the tissue and facilitate the extraction. In order to ensure we had enough material for our analysis, the liver specimens were pooled together to obtain 5 samples from each treatment group. The analysis was performed using an analytical High-Performance Liquid Chromatography with Fluorescence Detection (HPLC-FLD) method, which was optimized as detailed below. Following the optimized conditions, 1 g of each sample was extracted with 5 mL of a mixture containing acetonitrile/water (80/20, *v*/*v*). The extracts were then sonicated for 10 min, shaken for 1 h at RT using an orbital shaker (KS 4000 i control, IKA, Staufen, Germany), and centrifuged (15 min, 4 °C, 15,000× *g*). The resulting supernatants were diluted with 20 mL of ammonium acetate buffer (0.1 M, pH 4.8) and incubated overnight with 25 μL of the β-glucuronidase/arylsulfatase (30/60 U/mL) solution. Then, the samples were adjusted to pH 7 and cleaned using AflaTest™ WB immunoaffinity (IMA) columns, which were selective for the main AFs (AFB1, AFB2, AFG1, AFG2, AFM1, AFM2), as well as their metabolites (AFQ1 and AFL). During the cleaning process, the samples were eluted through the IMA columns, which were then washed with 20 mL of milliQ^®^ water to remove impurities. AFs were recovered in a silanized amber vial using 2 mL of methanol. The eluate was then evaporated at 35 °C using nitrogen, and residual AF were reconstituted with 500 μL of a methanol/water (50:50, *v*/*v*) mixture. Finally, 100 μL of the sample was injected into the HPLC system for the chromatographic analysis.

#### 5.6.3. HPLC–FLD Analysis of AF

The analytical method for the determination of AF in broiler liver samples was validated in-house according to the EC Regulation 519/2014 and the AOAC Requirements for Single Laboratory Validation of Chemical Methods (AOAC, 2016). Very high selectivity and specificity were guaranteed by the clean-up step, with the AflaTest^®^ WB IMA columns containing specific antibodies for AF, which provided chromatograms without any interference. The determination of AF was carried out using an Agilent 1100 HPLC system coupled with a fluorometric detector. The chromatographic separation of the AF was achieved using a Luna^®^ PFP (2) column (150 × 4.6 mm i.d., 3 μm, 100 Å) preceded by a KrudKatcher Classic HPLC In-Line Filter, 2.0 μm (Phenomenex, Torrance, CA, USA). The column temperature was set at 30 °C. The mobile phase consisting of water and acetonitrile (70:30, *v*/*v*) was eluted (1 mL/min) in isocratic mode. After 20 min elution, the column was washed with 90% acetonitrile for 5 min, and thereafter, it was re-equilibrated with the starting solvent (water:acetonitrile, 70:30, *v*/*v*). The sample injection volume was 100 μL (full-loop mode). The detection of AF was achieved using a fluorescence detector set at 365 nm (λem.) and 435 nm (λex.). To enhance the fluorescence of AFM1, AFB1, and AFG1, post-column photochemical derivatization was applied by using the UVE™ system (LCTech, Dorfen, Germany), which involved using UV radiation at 254 nm. AF were eluted as follows: AFM2 (4.8 min), AFM1 (5.6 min), AFQ1 (6.2 min), AFG2 (7.3 min), AFB2 (8.6 min), AFG1 (9.3 min), AFB1 (11 min), and aflatoxicol (12.3 min) (Figure S2).

The linearity range of the method was tested by preparing 5 different standard solutions (calibrants) containing each AF in the 0.05−5 µg/mL concentration range, which corresponds to a contamination level ranging from 25 to 2500 ng/Kg (ppt) of liver. Matrix-matched calibration curves were also prepared. Five different matrix-matched calibrants (in the range 0.05−5 µg/mL) were obtained by spiking the cleaned-up extracts of blank liver samples in methanol with an appropriate amount of AF stock solution depending on the desired levels. Calibrants were prepared in water/methanol (50/50, *v*/*v*) and analyzed in triplicate. Coefficients of determination (R2) were calculated for the both standard and matrix-matched calibration graphs (Table S2). To assess any possible matrix-interfering effect on AF detection, the signal suppression/enhancement values (SSE%), defined as the percentage ratio of the matrix-matched calibration slope to the solvent calibration slope, were calculated and found to range between 91 and 107%, confirming the efficiency of the method in separating matrix-interfering compounds from target AF. The LOD (limit of detection) and LOQ (limit of quantification) values, expressed as ng of AF/Kg of liver, were estimated as the concentration of analyte which provided a signal-to-noise ratio (S/N) higher than 3 and 10, respectively. Table S3 summarizes the LOD and LOQ values for each analyte (i.e., AFB1, AFB2, AFG1, AFG2, AFM1, AFM2, AFQ1, AFL). The recovery values were calculated by spiking liver samples at 100 and 1000 ng/Kg, while the accuracy and precision (relative standard deviation, RSD%) of the method were determined by analyzing three replicates for each spiking level (Table S4). Except for AFM2 and AFQ1, recovery values were acceptable. The calculated RSD values were ≤9% for all toxins and were in the range of acceptability as defined by the EC Regulation 519/2014.

### 5.7. Statistics

Data were expressed as mean ± standard deviation (SD) or standard error of the mean (SEM), and the normal distribution of the data was assessed according to the D’Agostino and Pearson normality omnibus test (*n* = 8 for each experimental group). Significant differences among groups were evaluated by a one-way analysis of variance (ANOVA), followed by Tukey’s post hoc tests. Differences were considered statistically significant when the two-sided *p* value was <0.05. Data analysis was performed using GraphPad Prism 7.03 software (Graph Pad Software, San Diego, CA, USA).

## Figures and Tables

**Table 1:** Histopathological evaluation of liver tissues from experimental broilers.

Parameter	Treatment				*p*-Value
	Ctrl	TP	AFB1	AFB1 + TP	
Vacuolar degeneration	Absence of alterations				
Lymphoplasmacytic Inflammation	1.000 (0.6–1.3)	0.500 (0.0–0.6)	0.500 (0.0–0.5)	1.000 (0.5–1.0)	0.117

Ctrl, control group; TP, turmeric powder group; AFB1, Aflatoxin B1 group; AFB1 + TP, Aflatoxin B1 plus turmeric powder group. Data are expressed as mean and interquartile range. The *p*-value tab refers to the results of a one-way ANOVA among all the experimental groups.

**Table 2:** GSH content and enzymatic activities of total GST, GST α, and GST µ in liver samples from experimental broilers.

Parameter	Treatment			
	Ctrl	TP	AFB1	AFB1 + TP
GSH (µmol/g liver)	2.23 ± 0.75	2.56 ± 0.42	2.15 ± 0.54	2.43 ± 0.25
Total GST (nmol/min/mg protein)	504 ± 85	548 ± 123	563 ± 83	493 ± 83
GST α (nmol/min/mg protein)	45.1 ± 9.0	40.7 ± 8.7	40.3 ± 5.7	36.8 ± 3.3
GST µ (nmol/min/mg protein)	1.04 ± 0.19	1.30 ± 0.48	1.50 ± 0.69	1.47 ± 0.66

Ctrl, control group; TP, turmeric powder group; AFB1, Aflatoxin B1 group; AFB1 + TP, Aflatoxin B1 plus turmeric powder group. Data are expressed as mean ± SD.

**Table 3:** Content of AFB1 in broiler liver as measured by an in-house-validated HPLC–FLD method.

Pooled Sample	Treatment		
	Ctrl	AFB1	AFB1 + TP
1	<LOD	26.0	<LOD
2	<LOD	37.0	<LOD
3	29.3	81.0	<LOD
4	20.8	49.3	<LOD
5	<LOD	36.1	<LOD
Mean ± SEM	10.2 ± 5.6	45.9 ± 8.5	-

Ctrl, control group; AFB1, Aflatoxin B1 group; AFB1 + TP, Aflatoxin B1 plus turmeric powder group. Data are expressed as ng/kg. LOD = 8 ng/kg.

## Data Availability

The raw data supporting the findings of this manuscript will be made available by the corresponding author, C.N., or the last author, F.G., to any researcher upon reasonable request.

## Supplementary Materials

The following supporting information can be downloaded at: https://www.mdpi.com/article/10.3390/toxins15120687/s1; Table S1: Primers for quantitative real-time PCR (qRT-PCR); Table S2: Matrix-matched regression curves and related R2 and SSE (%) values; Table S3: Limit of detection (LOD, S/N = 3) and limit of quantification (LOQ, S/N = 10) calculated for AFB1, AFB2, AFG1, AFG2, AFM1, AFM2, AFQ1, and AFL in liver samples; Table S4: Recoveries of AFB1, AFB2, AFG1, AFG2, AFM1, AFM2, AFQ1, and AFL in liver samples spiked at two different AFBs levels (0.1 and 1 ng/g). Figure S1: qRT-PCR dissociation curves for each gene in liver (L) and intestine (I); Figure S2: HPLC-FLD chromatograms of AF obtained using the analytical conditions detailed in Section 5.6.3. The red chromatogram represents a standard calibrant of AF, the green and blue chromatograms are those of liver samples belonging to the AFB1 group and AFB1 plus TP group, respectively.
