# Supplementary material for: Turmeric Powder Counteracts Oxidative Stress and Reduces AFB1 Content in the Liver of Broilers Exposed to the EU Maximum Levels of the Mycotoxin
Source: Toxins (Basel). 2023 Dec 7;15(12):687. doi: 10.3390/toxins15120687 (PMC10747922; doi:10.3390/toxins15120687)
Supplement: Supplementary file 1 [file toxins-15-00687-s001.zip › toxins-2731337-supplementary Figure S1.pdf]

Figure S1. qRT-PCR **d**issociation curves for each gene in liver (L) and intestine (I).

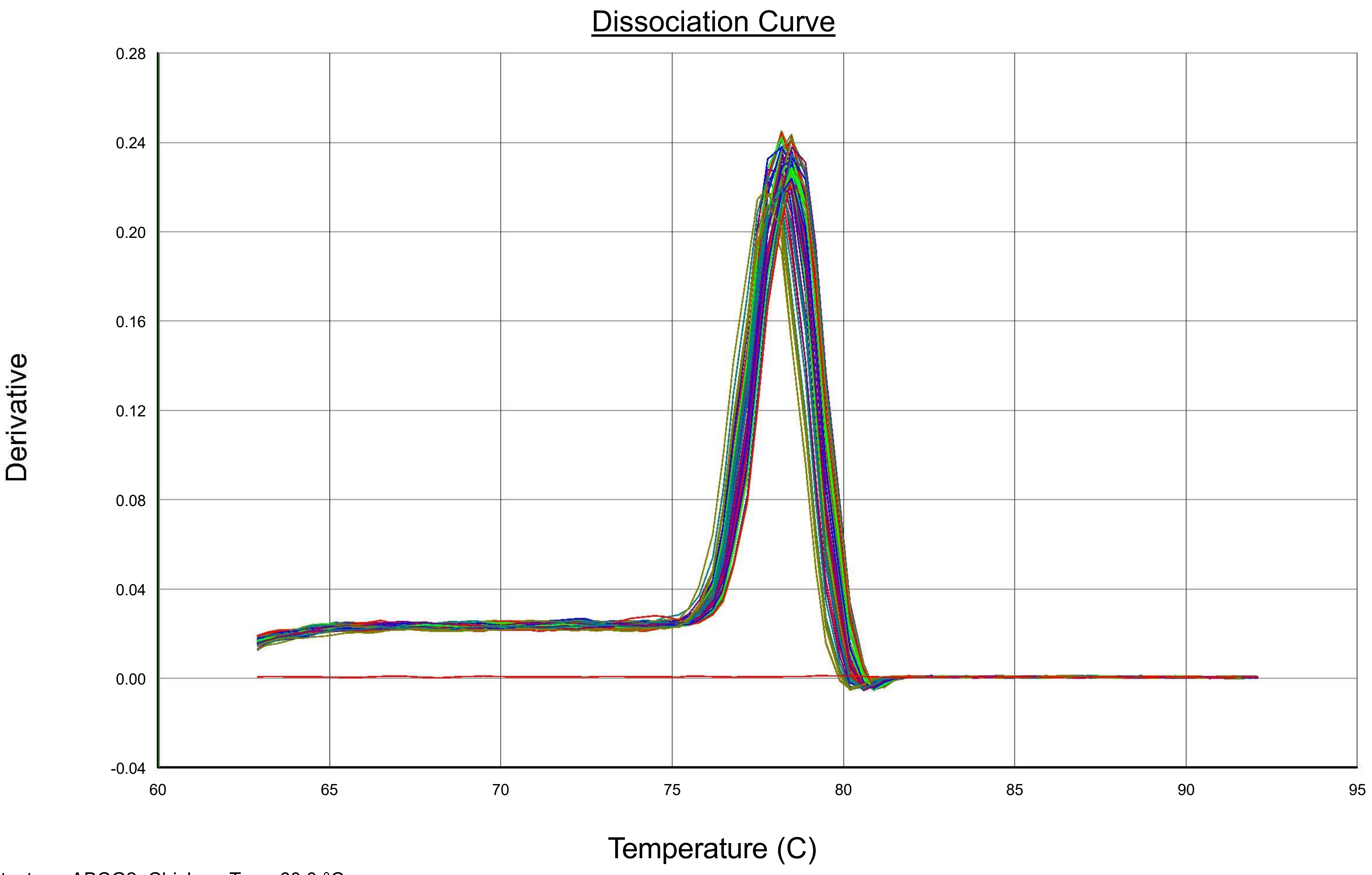

Detector = ABCG2_Chicken, Tm = 60.0 °C
Well(s): A1-D12,H7-H8
Document: L_Broiler_ABCG2_plate 1 (Absolute Quantification)

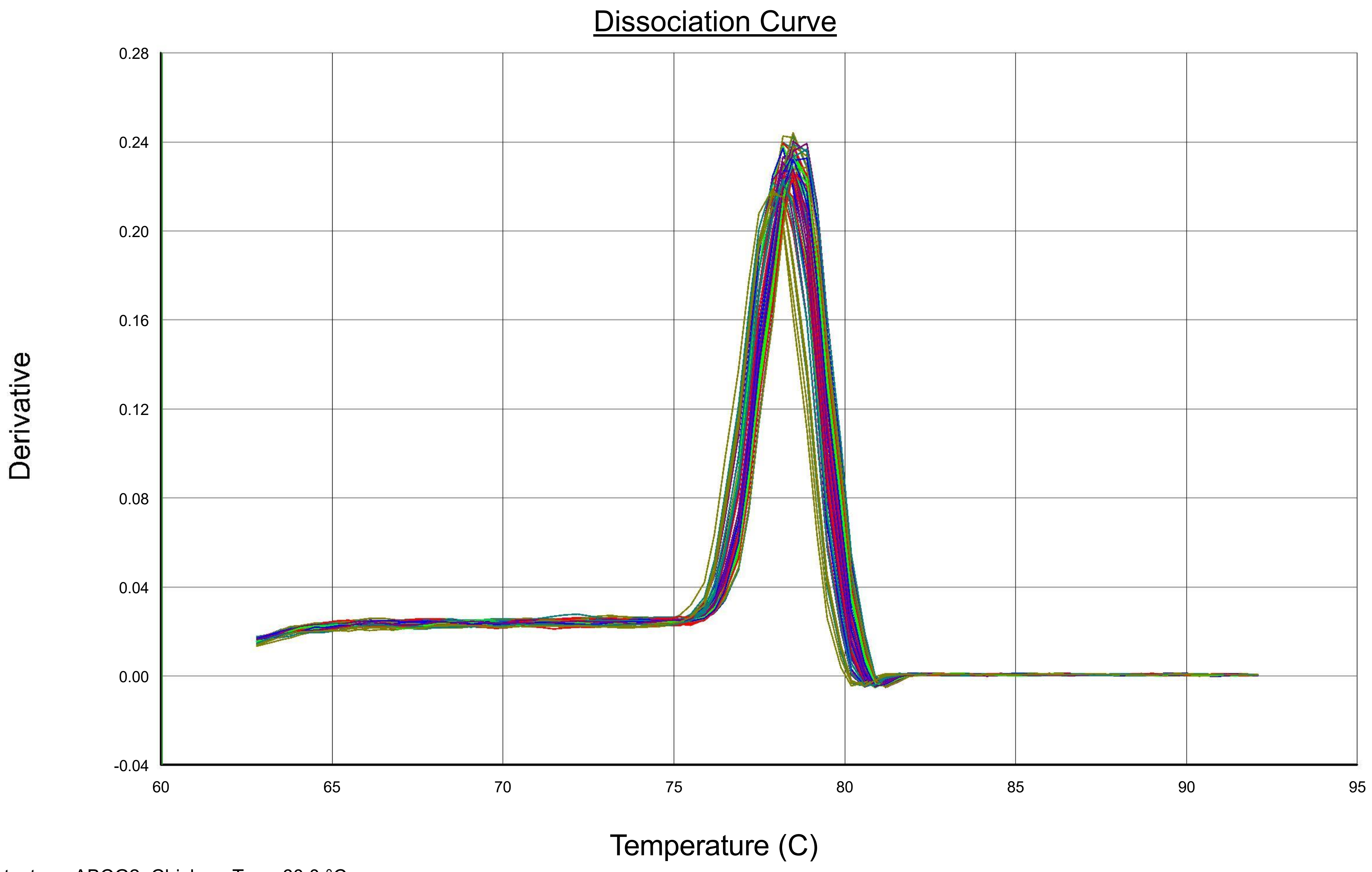

Detector = ABCG2_Chicken, Tm = 60.0 °C
Well(s): A1-D12
Document: L_Broiler_ABCG2_plate 2 (Absolute Quantification)

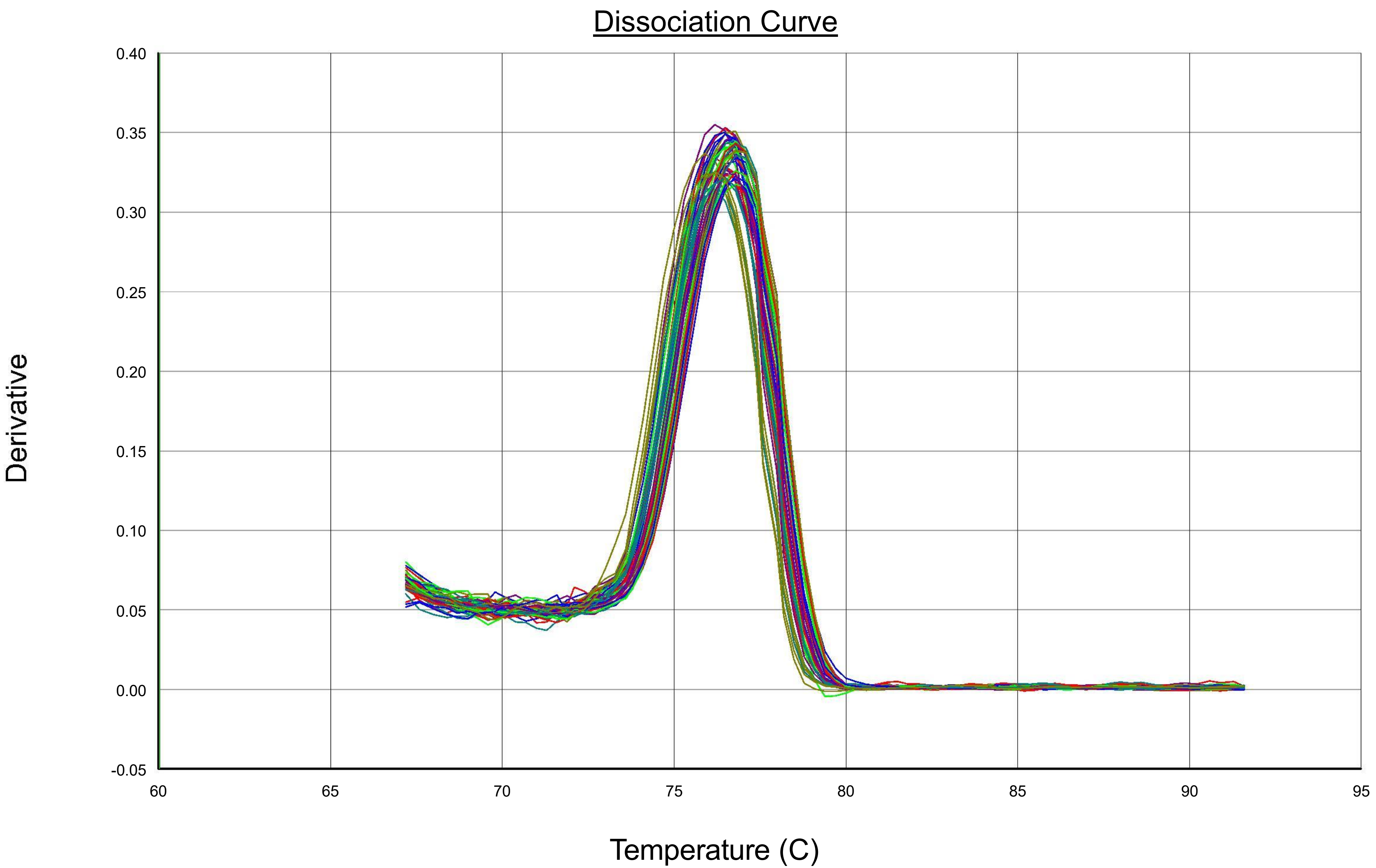

Detector = CAT_Chicken, Tm = 60.0 °C
Well(s): A1-D12
Document: L_Broiler_CAT_plate 1 (Absolute Quantification)

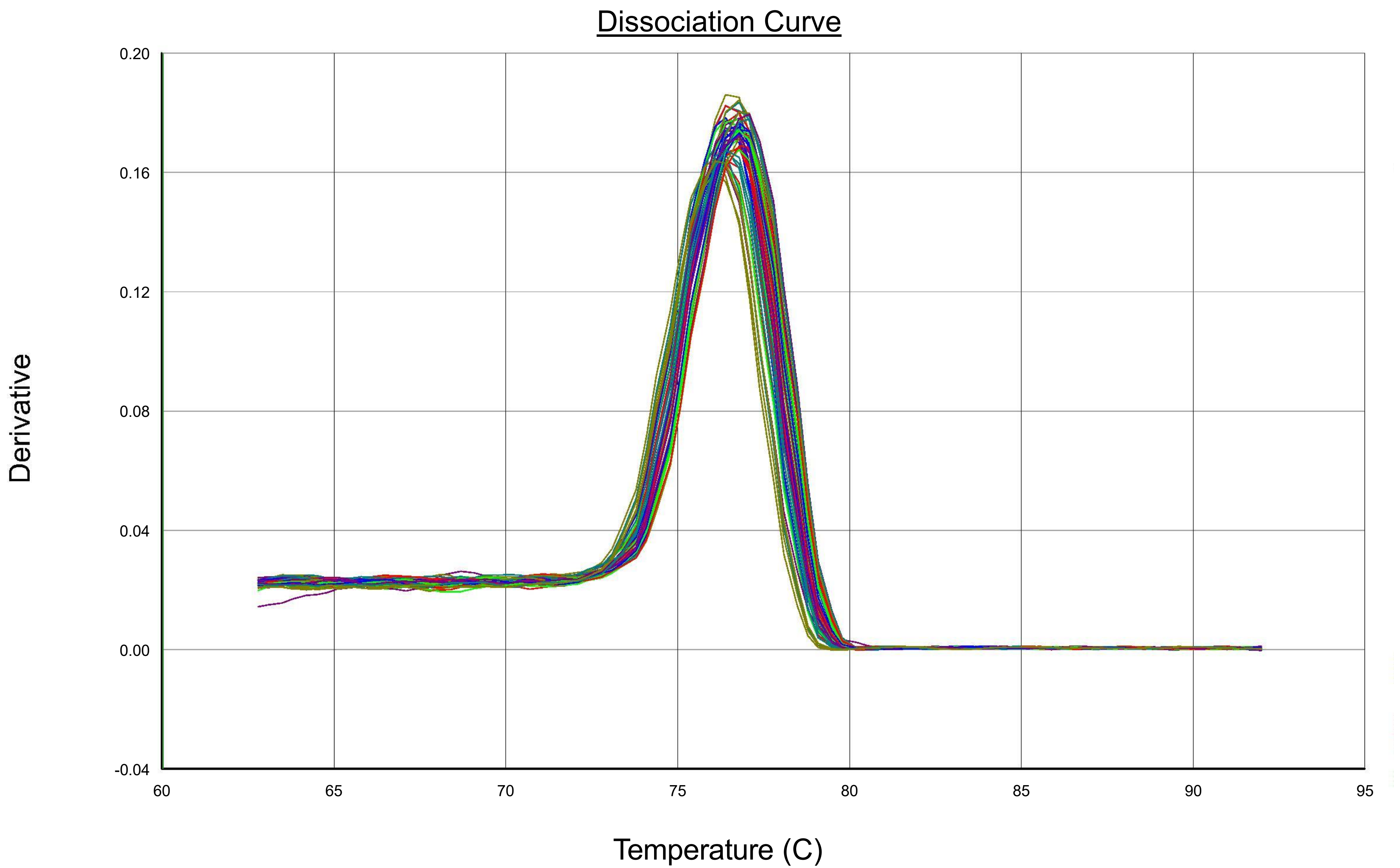

Detector = CAT_Chicken, Tm = 60.0 °C

Well(s): A1-D12

Document: L_Broiler_CAT_plate 2 (Absolute Quantification)

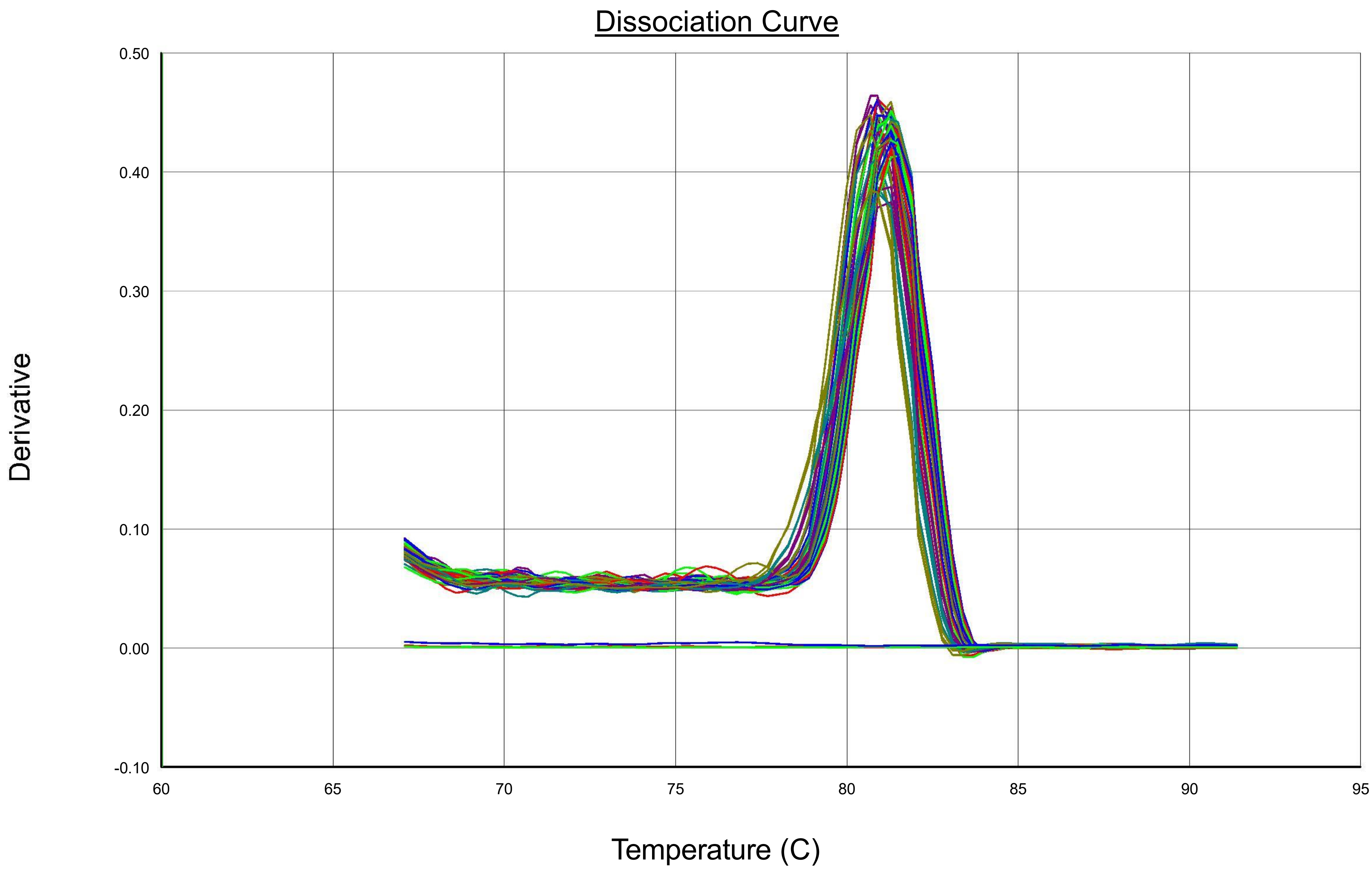

Detector = CYP2A6, Tm = 60.0 °C
Well(s): A1-D12,H7-H9
Document: L_Broiler_CYP2A6_plate 1.sds (Absolute Quantification)

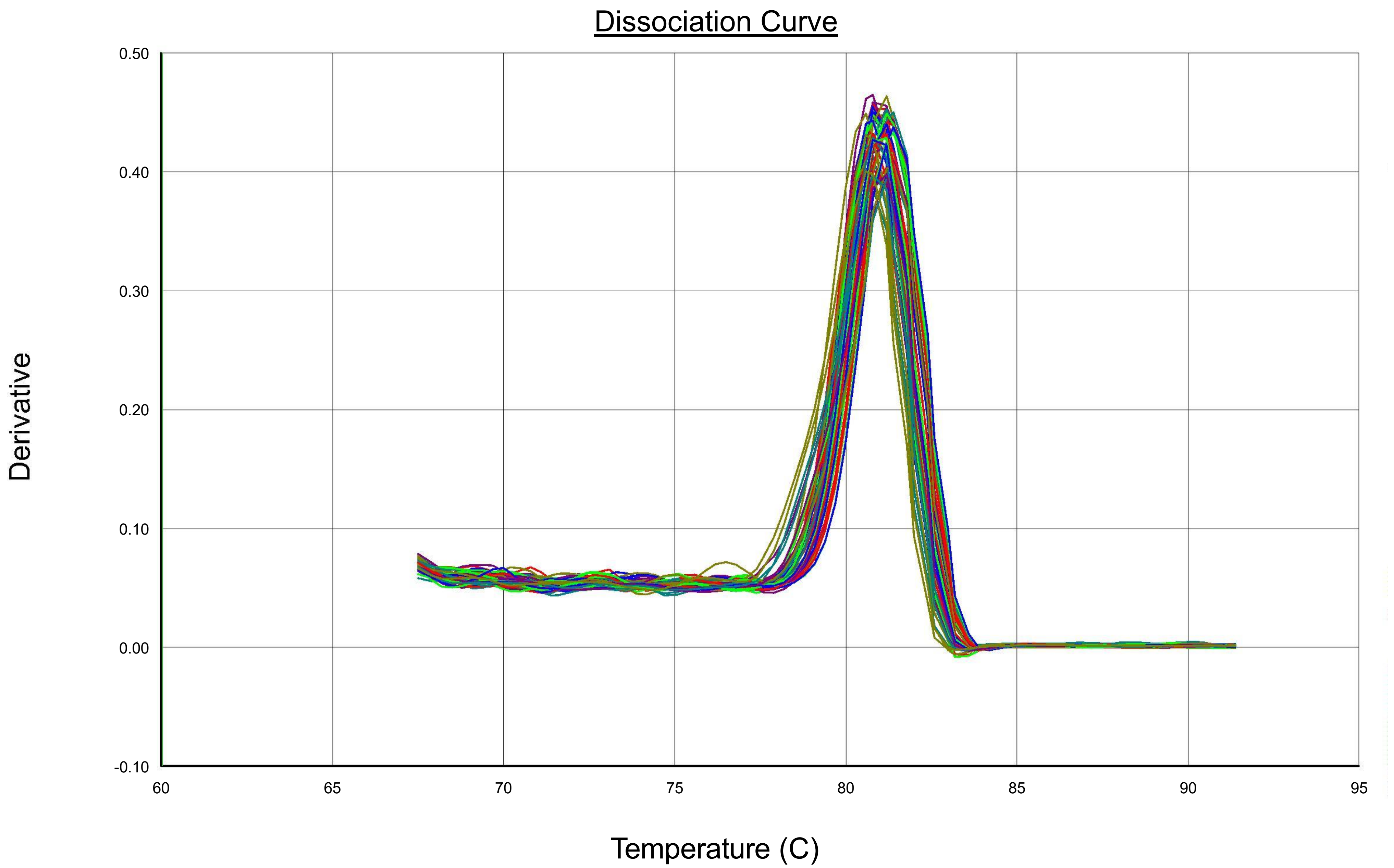

Detector = CYP2A6, Tm = 60.0 °C
Well(s): A1-D12
Document: L_Broiler_CYP2A6_plate 2.sds (Absolute Quantification)

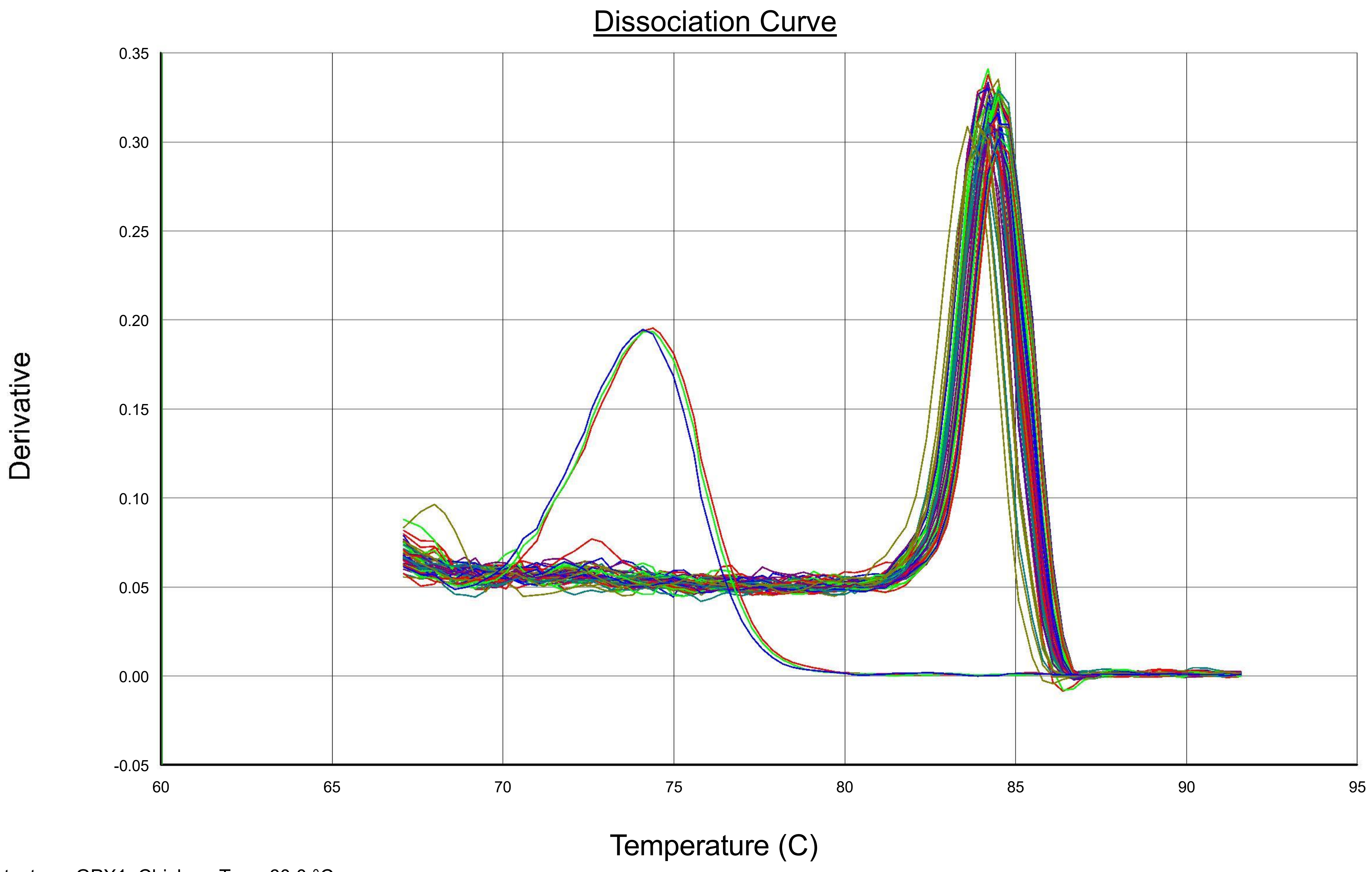

Detector = GPX1_Chicken, Tm = 60.0 °C
Well(s): A1-D12,H7-H9
Document: L_Broiler_GPX1_plate 1 (Absolute Quantification)

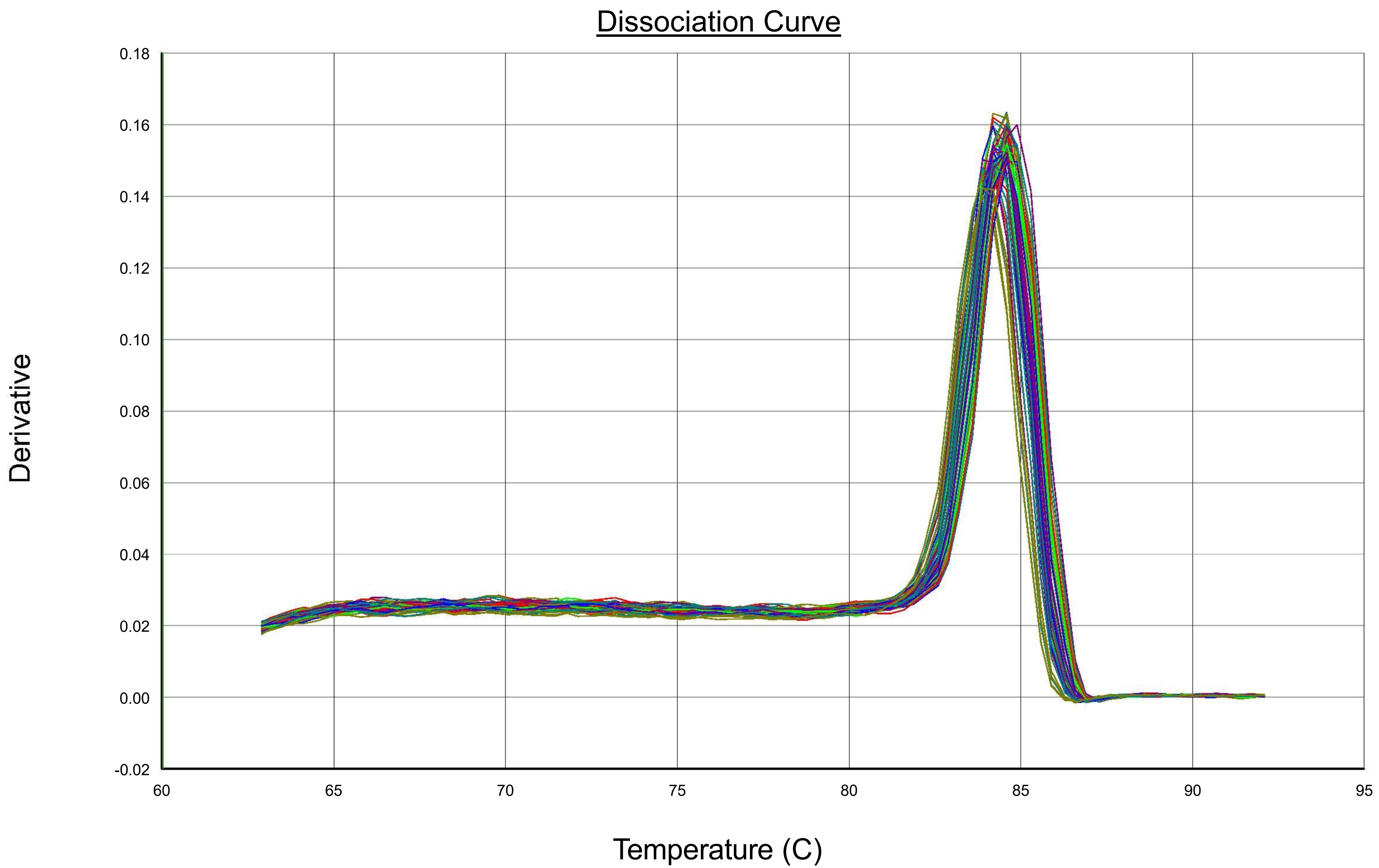

Detector = GPX1_Chicken, Tm = 60.0 °C

Well(s): A1-D12

Document: L_Broiler_GPX1_plate 2 (Absolute Quantification)

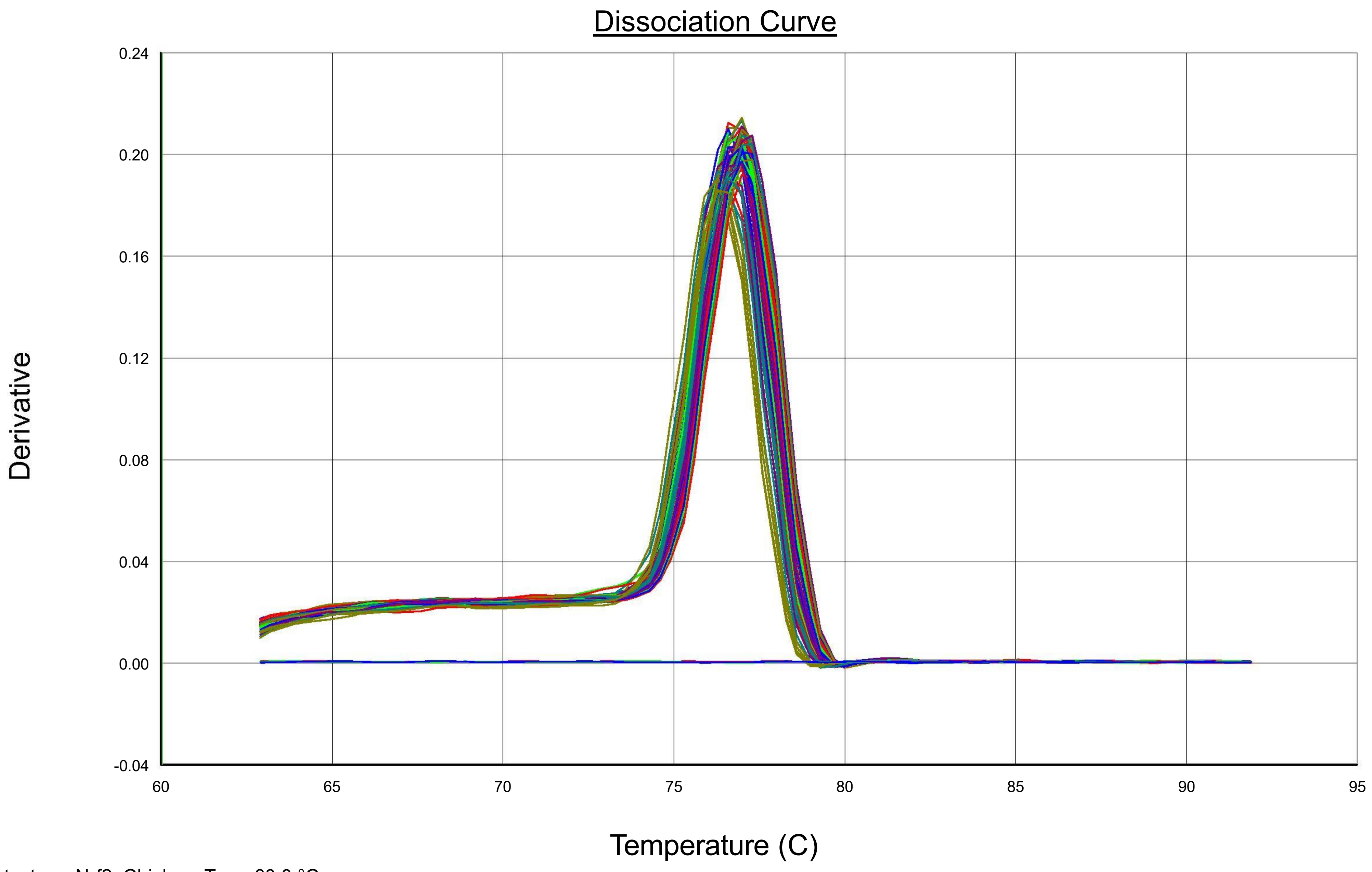

Detector = Nrf2_Chicken, Tm = 60.0 °C
Well(s): A1-D12,H7-H9
Document: L_Broiler_Nrf2_plate1.sds (Absolute Quantification)

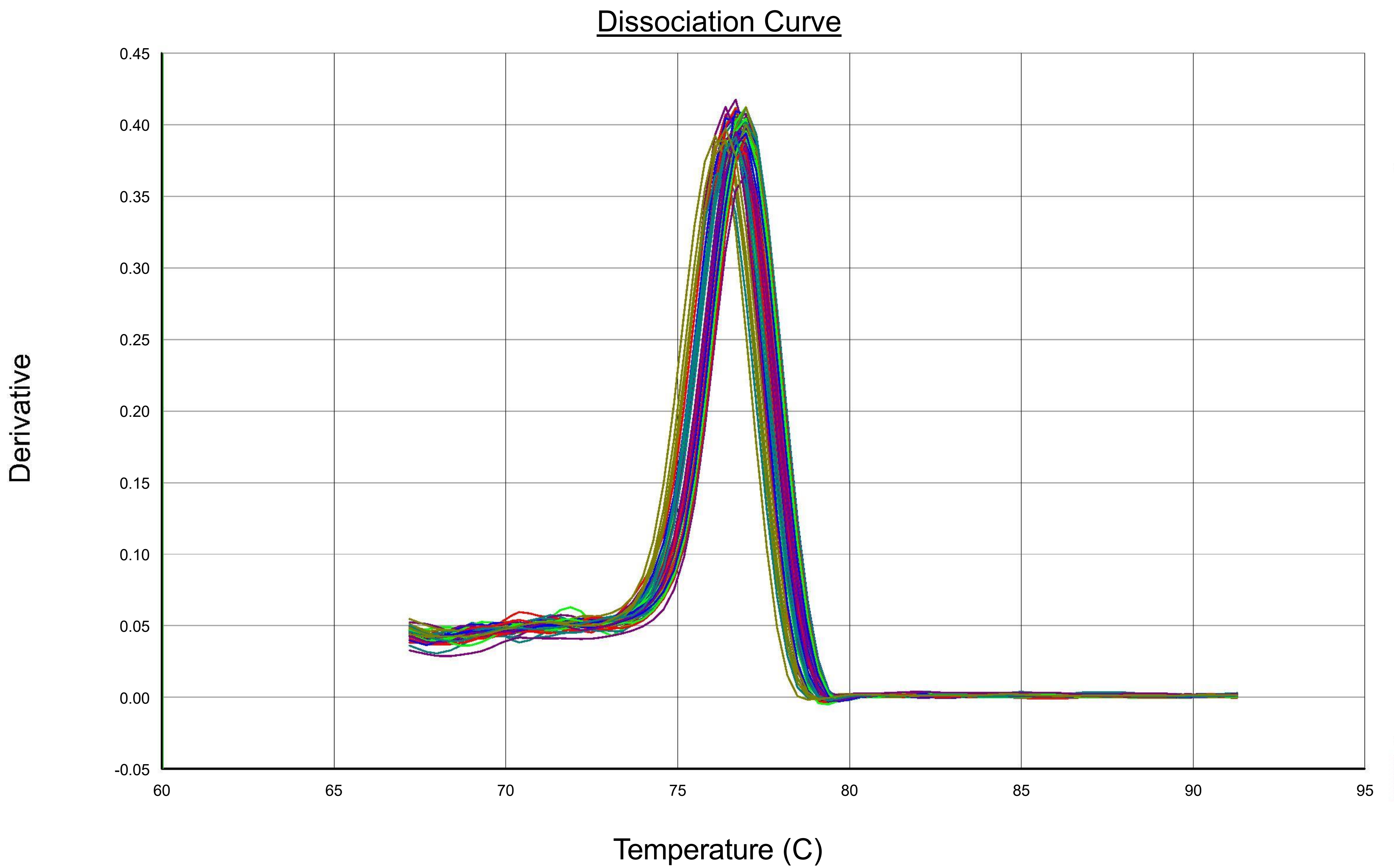

Detector = Nrf2_Chicken, Tm = 60.0 °C
Well(s): A1-D12
Document: L_Broiler_Nrf2_plate2.sds (Absolute Quantification)

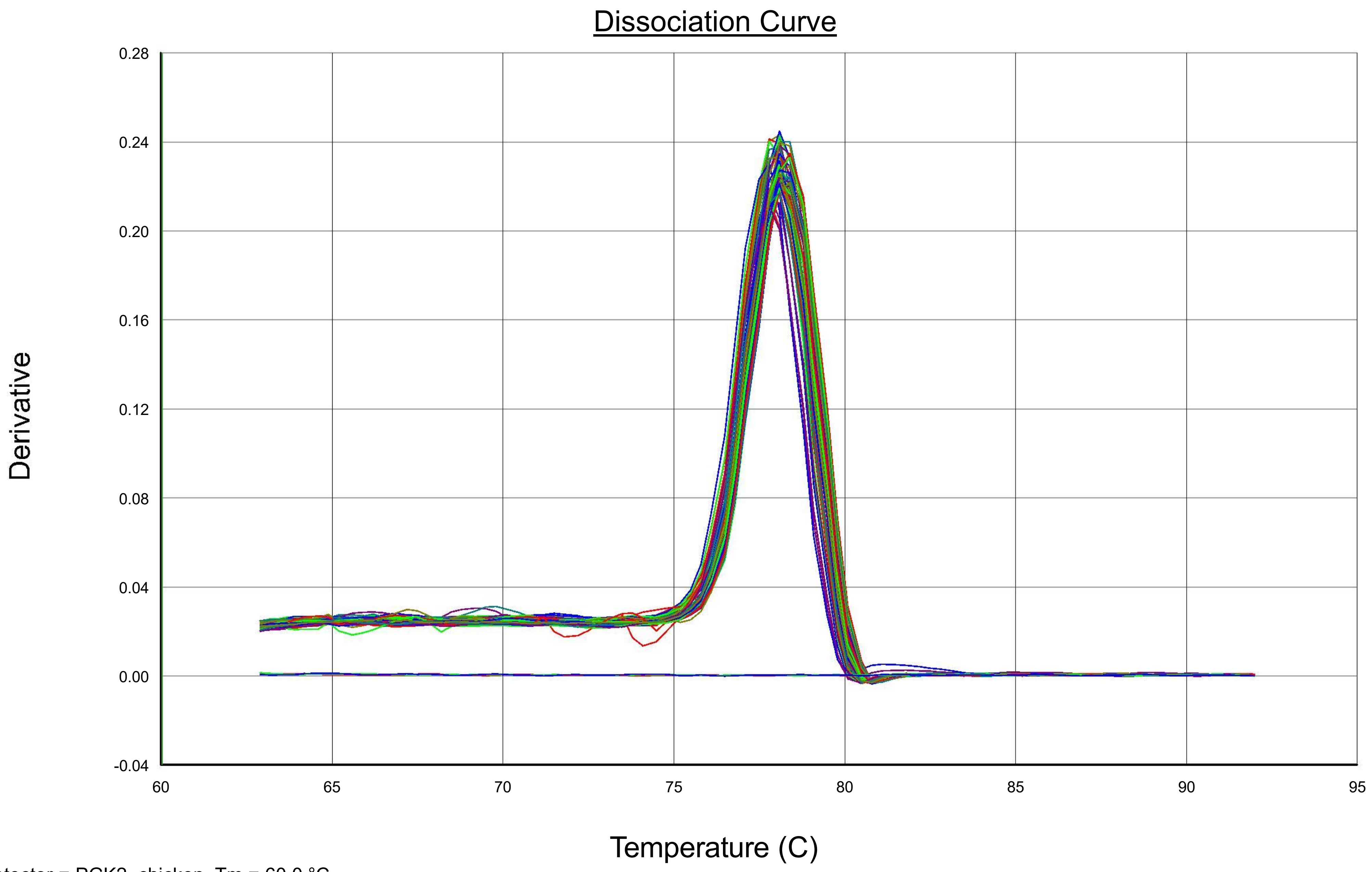

Detector = PGK2_chicken, Tm = 60.0 °C
Well(s): A1-D12,H10-H12
Document: L_Broiler_PGK2_plate 1 (Absolute Quantification)

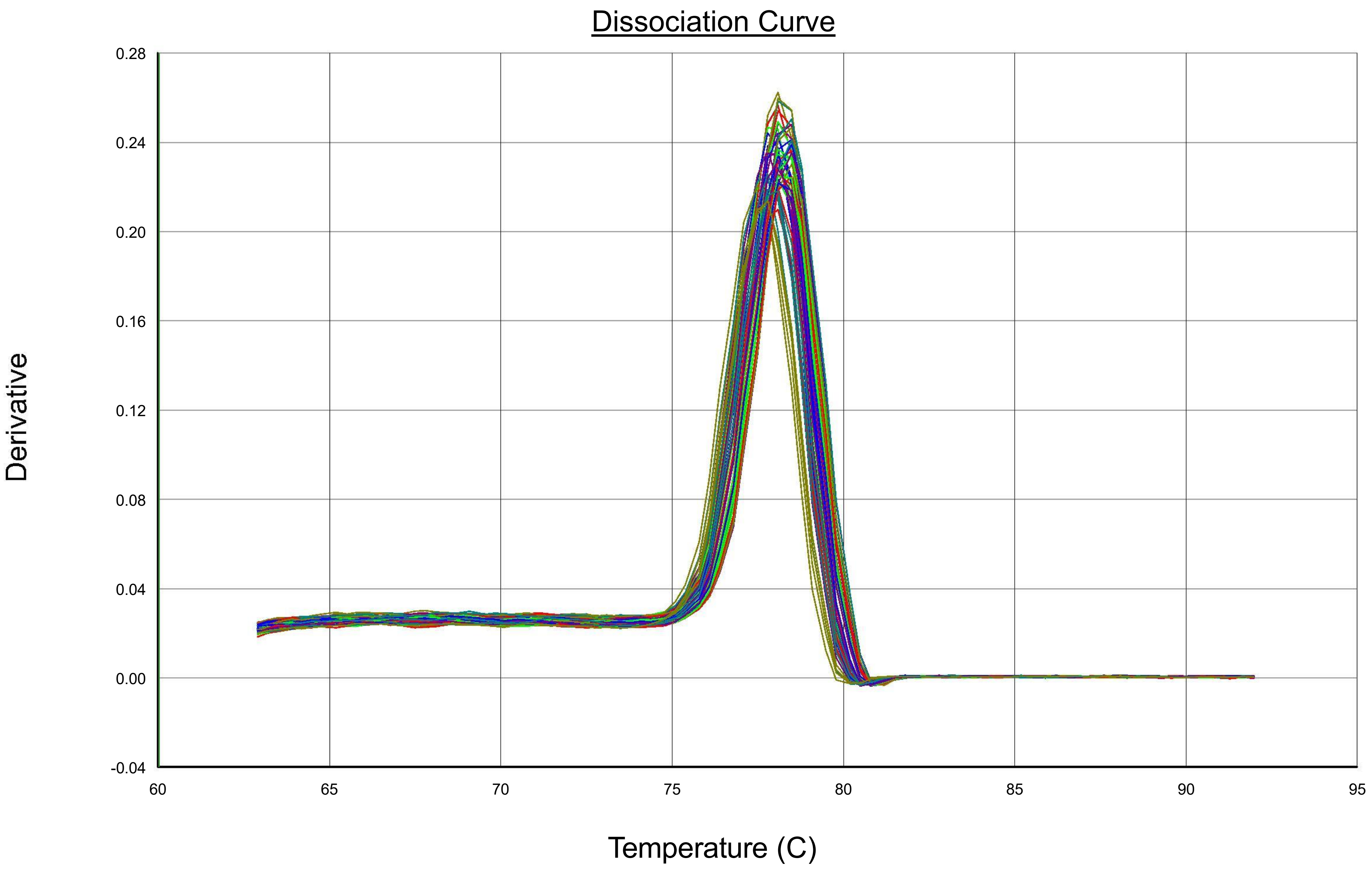

Detector = PGK2_chicken, Tm = 60.0 °C
Well(s): A1-D12
Document: L_Broiler_PGK2_plate 2 (Absolute Quantification)

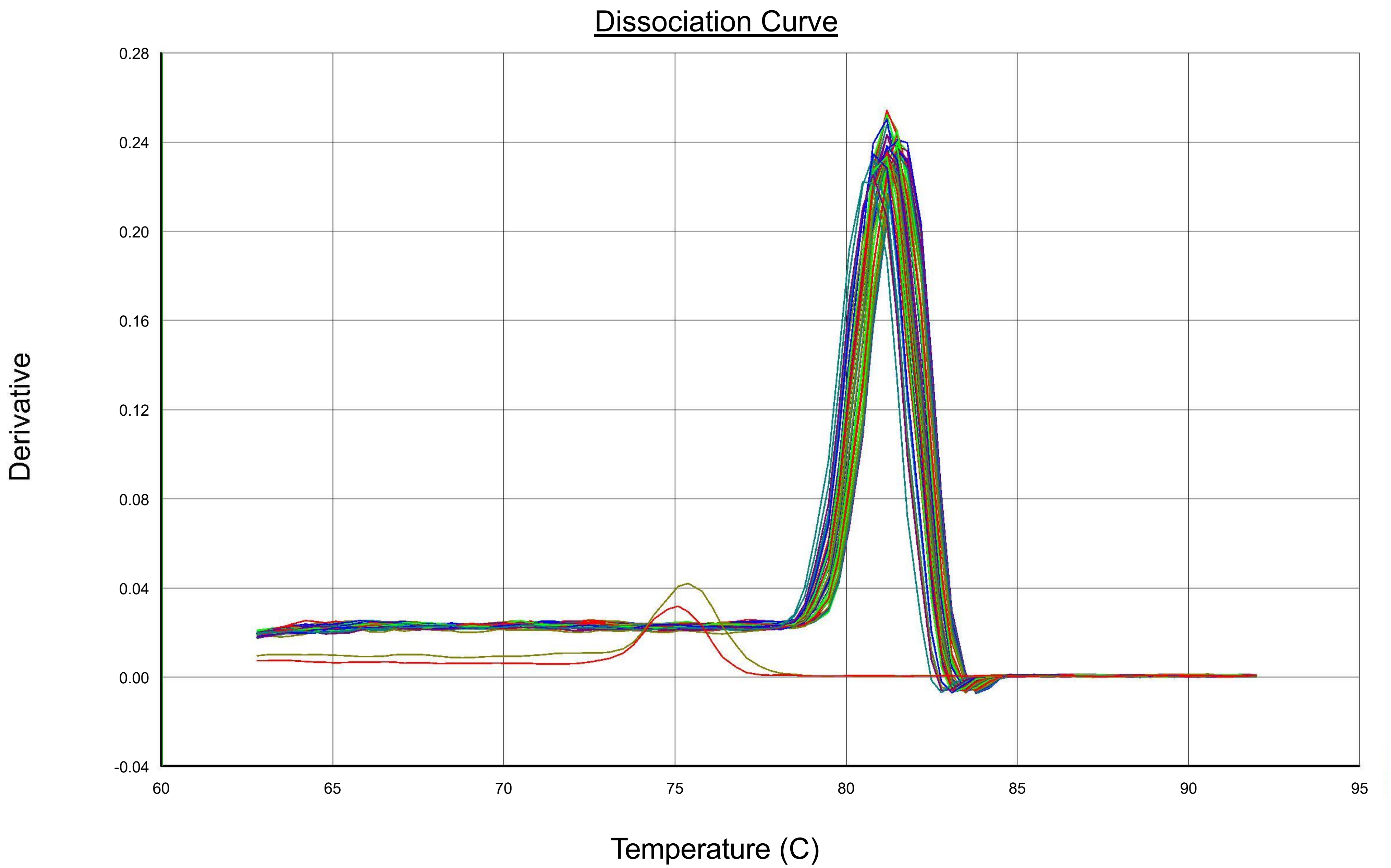

Detector = RPS7_Chicken, Tm = 60.0 °C
Well(s): A1-D12,H8-H9
Document: L_Broiler_RPS7_plate 1 (Absolute Quantification)

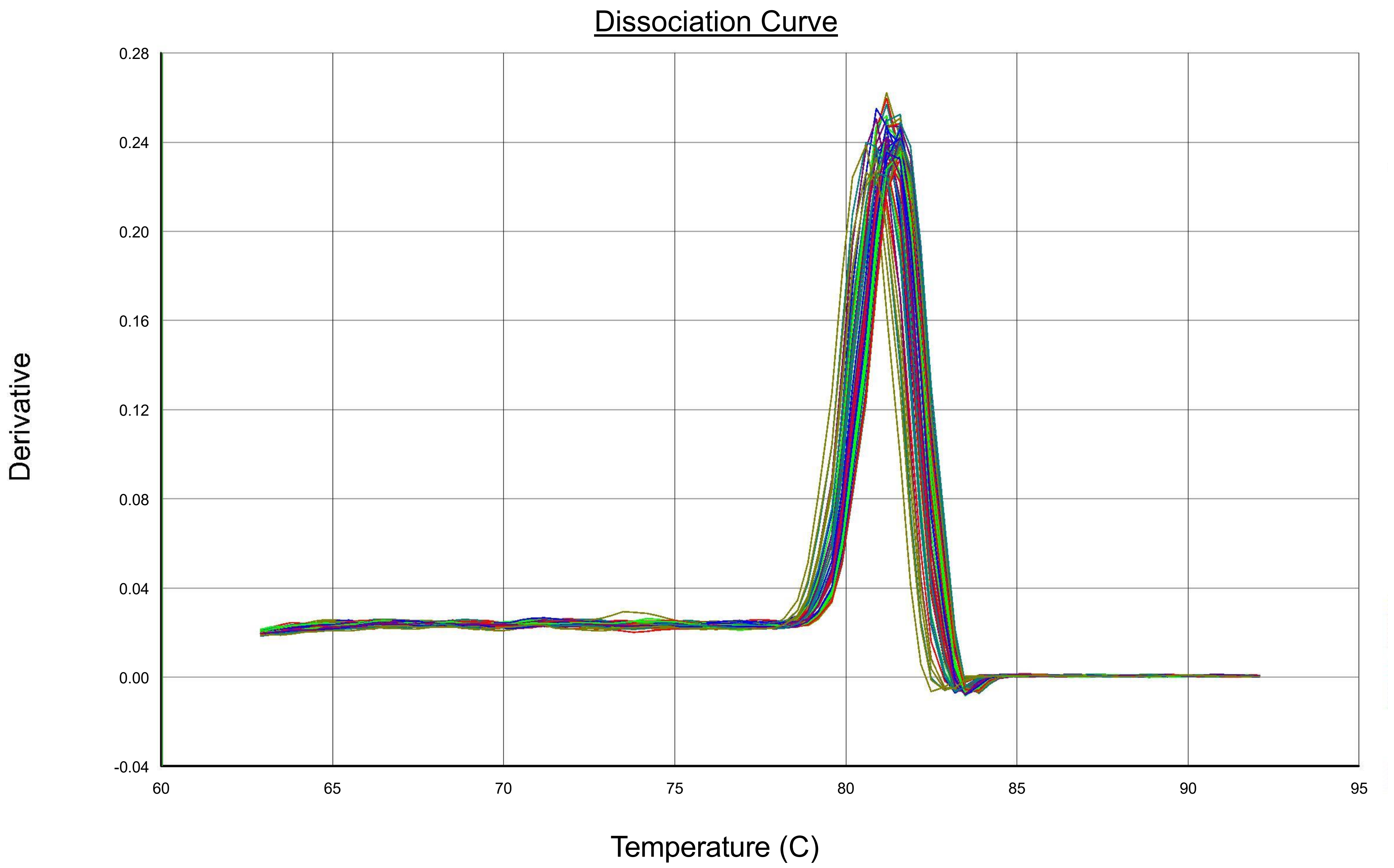

Detector = RPS7_Chicken, Tm = 60.0 °C
Well(s): A1-D12
Document: L_Broiler_RPS7_plate 2 (Absolute Quantification)

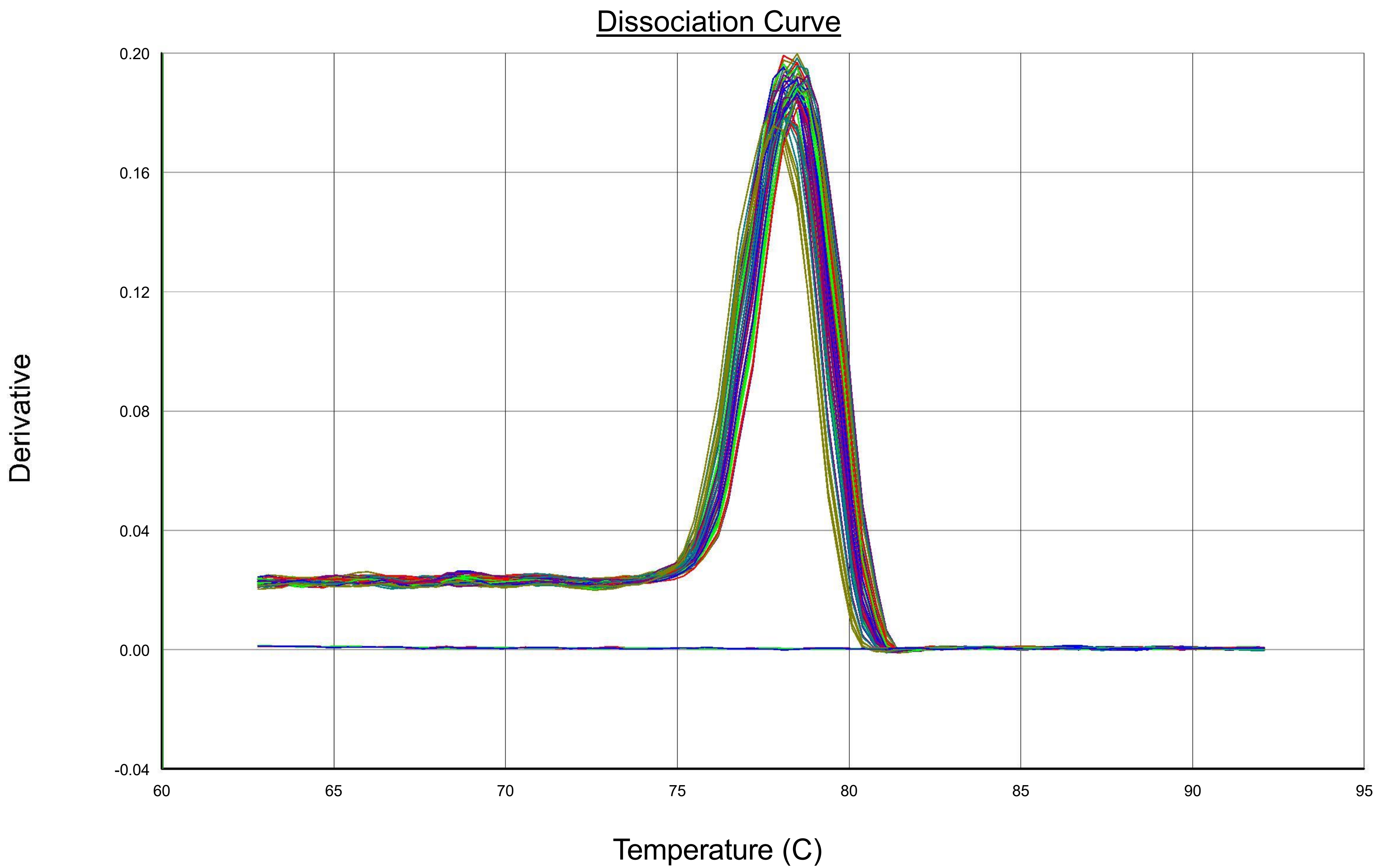

Detector = SOD2_Chicken, Tm = 60.0 °C
Well(s): A1-D12,H7-H9
Document: L_Broiler_SOD2_plate 1 (Absolute Quantification)

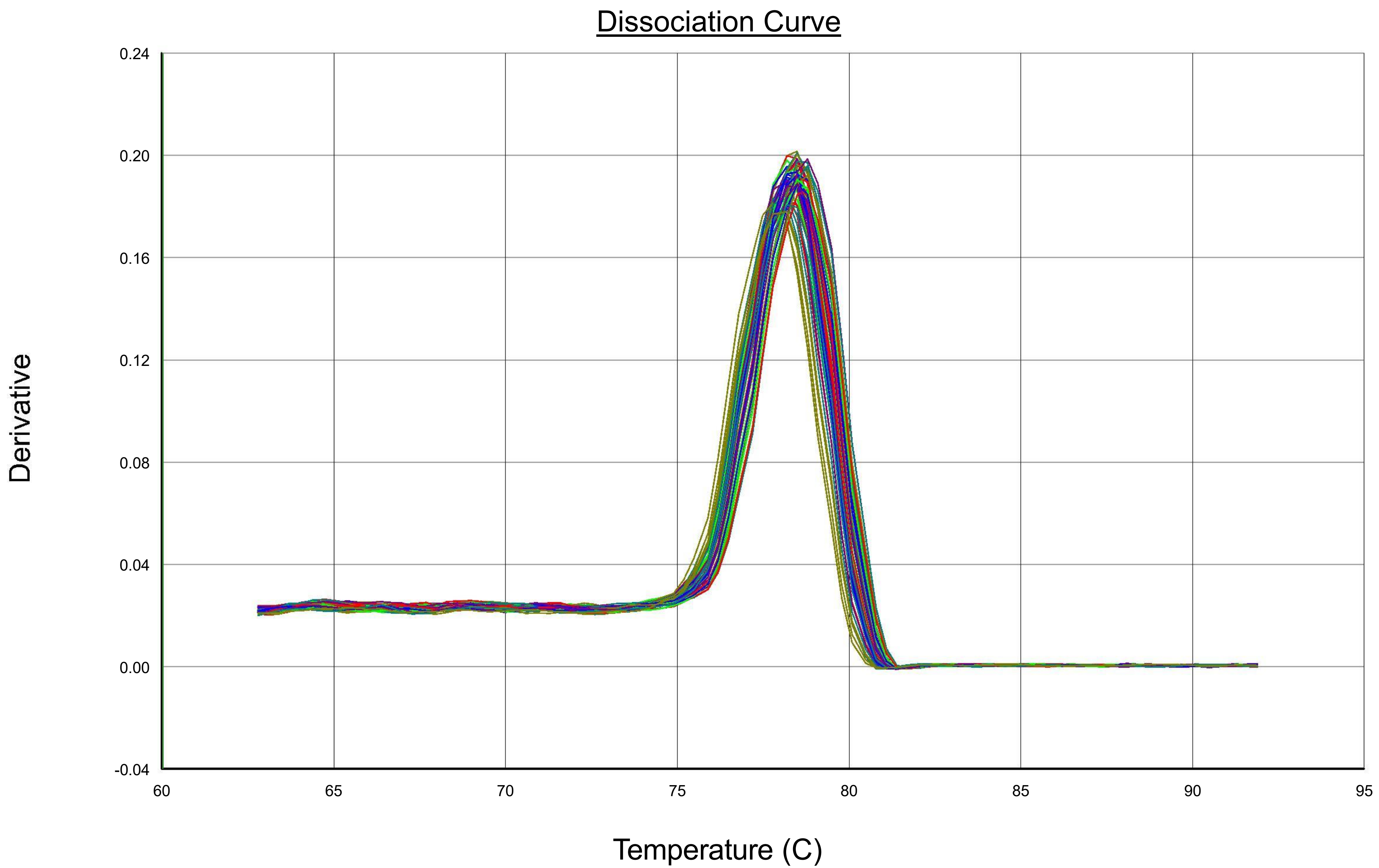

Detector = SOD2_Chicken, Tm = 60.0 °C
Well(s): A1-D12
Document: L_Broiler_SOD2_plate 2 (Absolute Quantification)

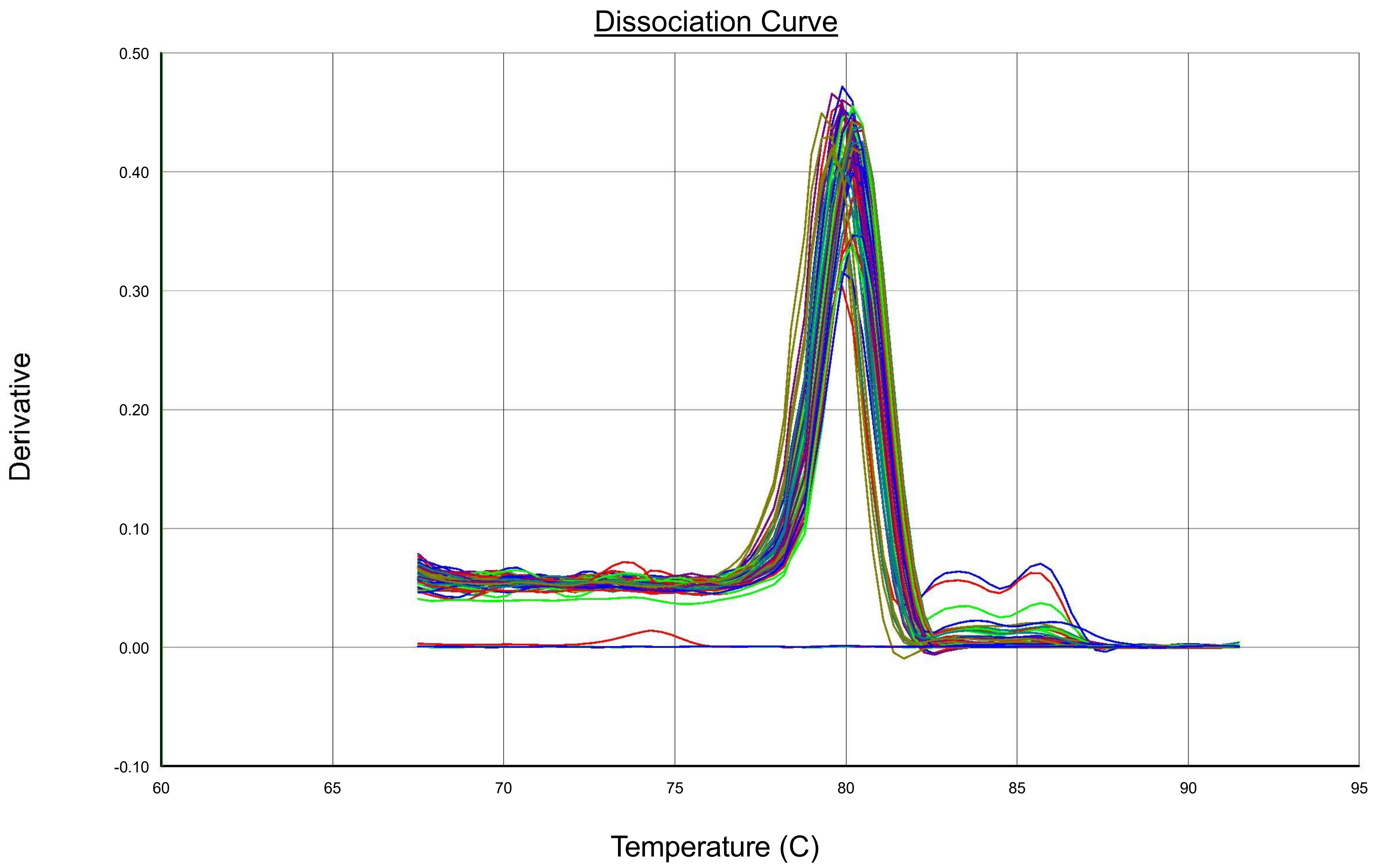

Detector = ABCC2_Chicken, Tm = 60.0 °C
Well(s): A1-D12,H7-H9
Document: I_Broiler_ABCC2_plate 1.sds (Absolute Quantification)

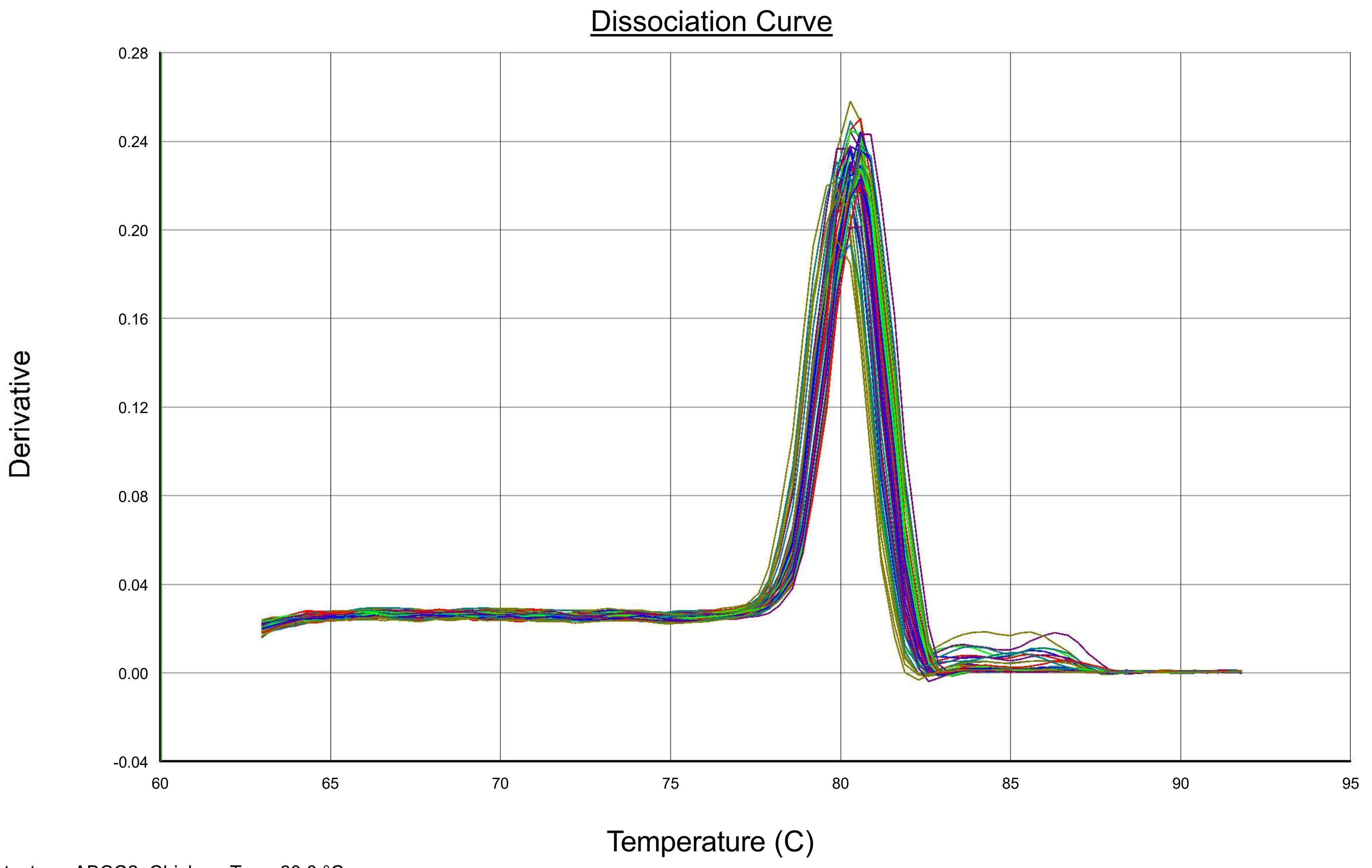

Detector = ABCC2_Chicken, Tm = 60.0 °C
Well(s): A1-D12
Document: I_ABCC2_plate 2 (Absolute Quantification)

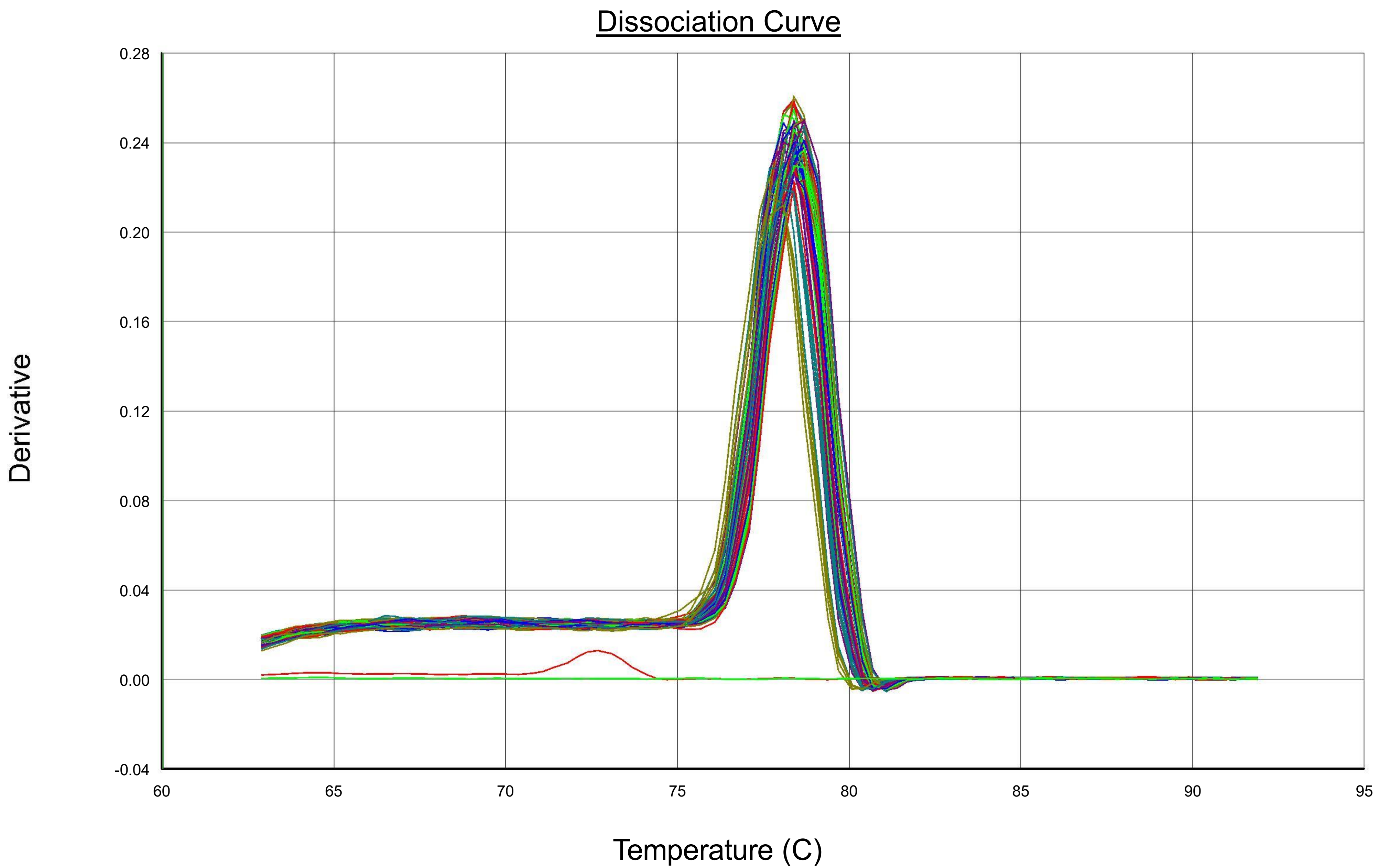

Detector = ABCG2_Chicken, Tm = 60.0 °C
Well(s): A1-D12,H8-H9
Document: I_ABCG2_plate 1 (Absolute Quantification)

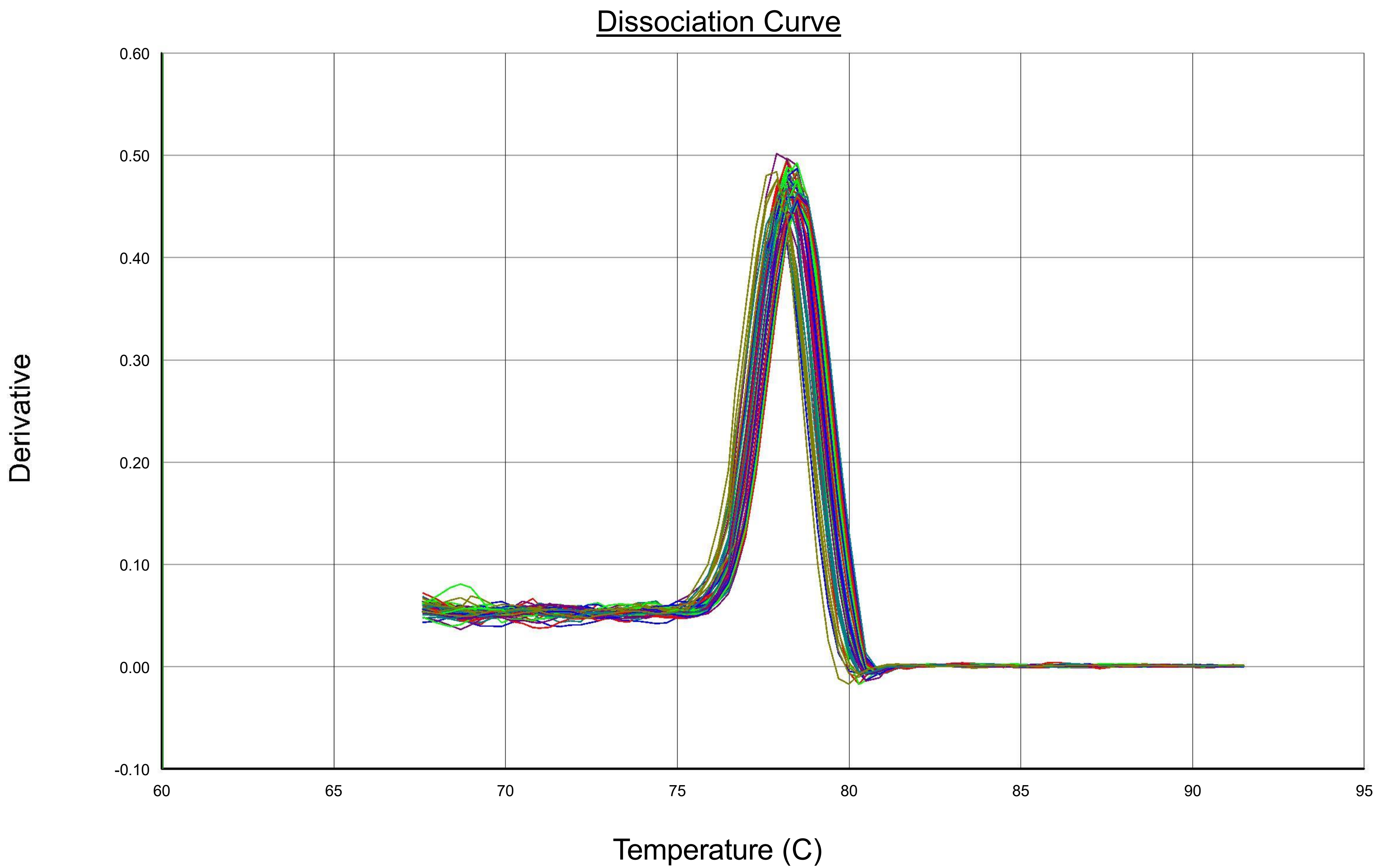

Detector = ABCG2, Tm = 60.0 °C
Well(s): A1-D12
Document: I_ABCG2_plate 2 (Absolute Quantification)

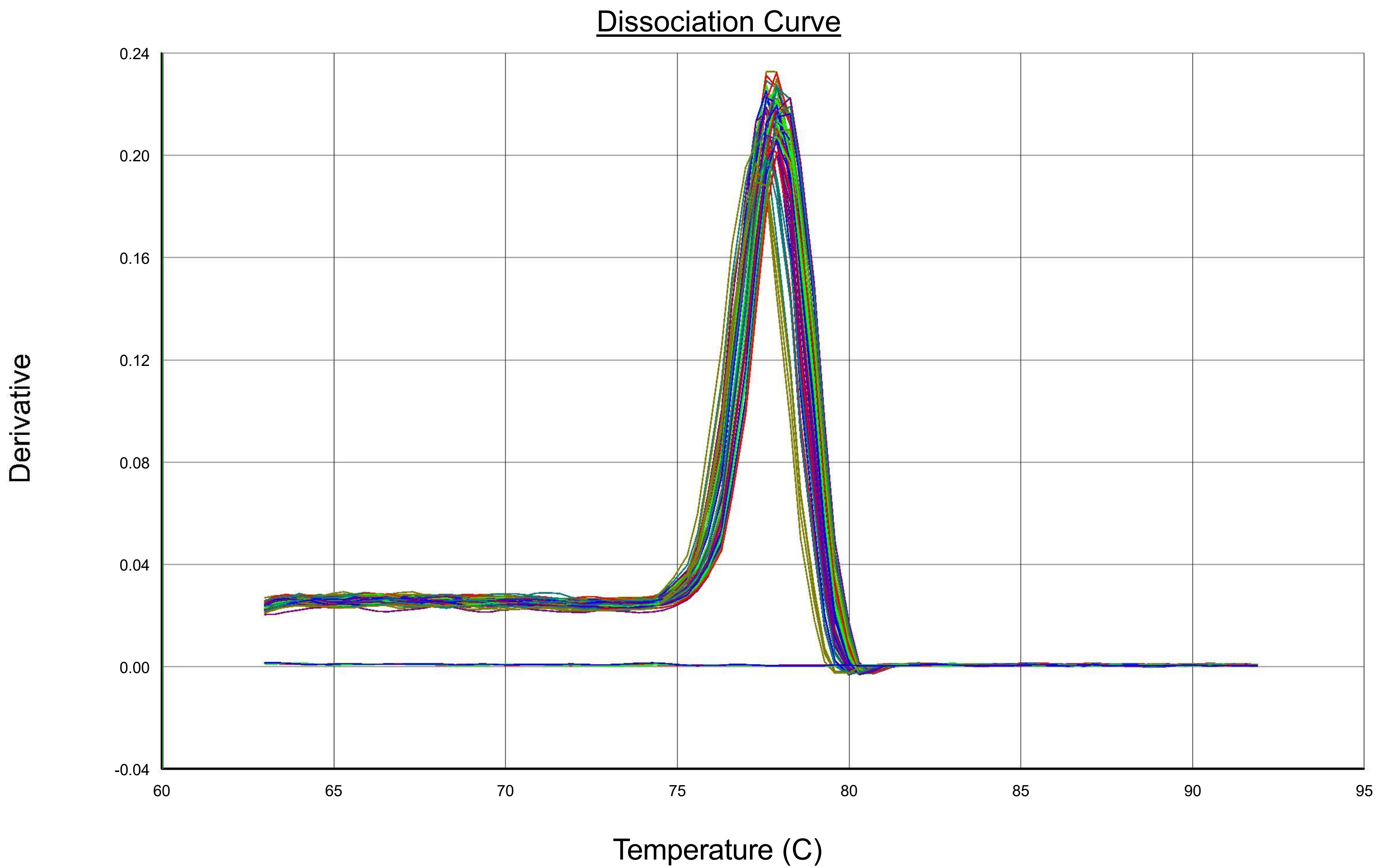

Detector = GUSB_chicken, Tm = 60.0 °C
Well(s): A1-D12,H7-H9
Document: I_GUSB_plate 1 (Absolute Quantification)

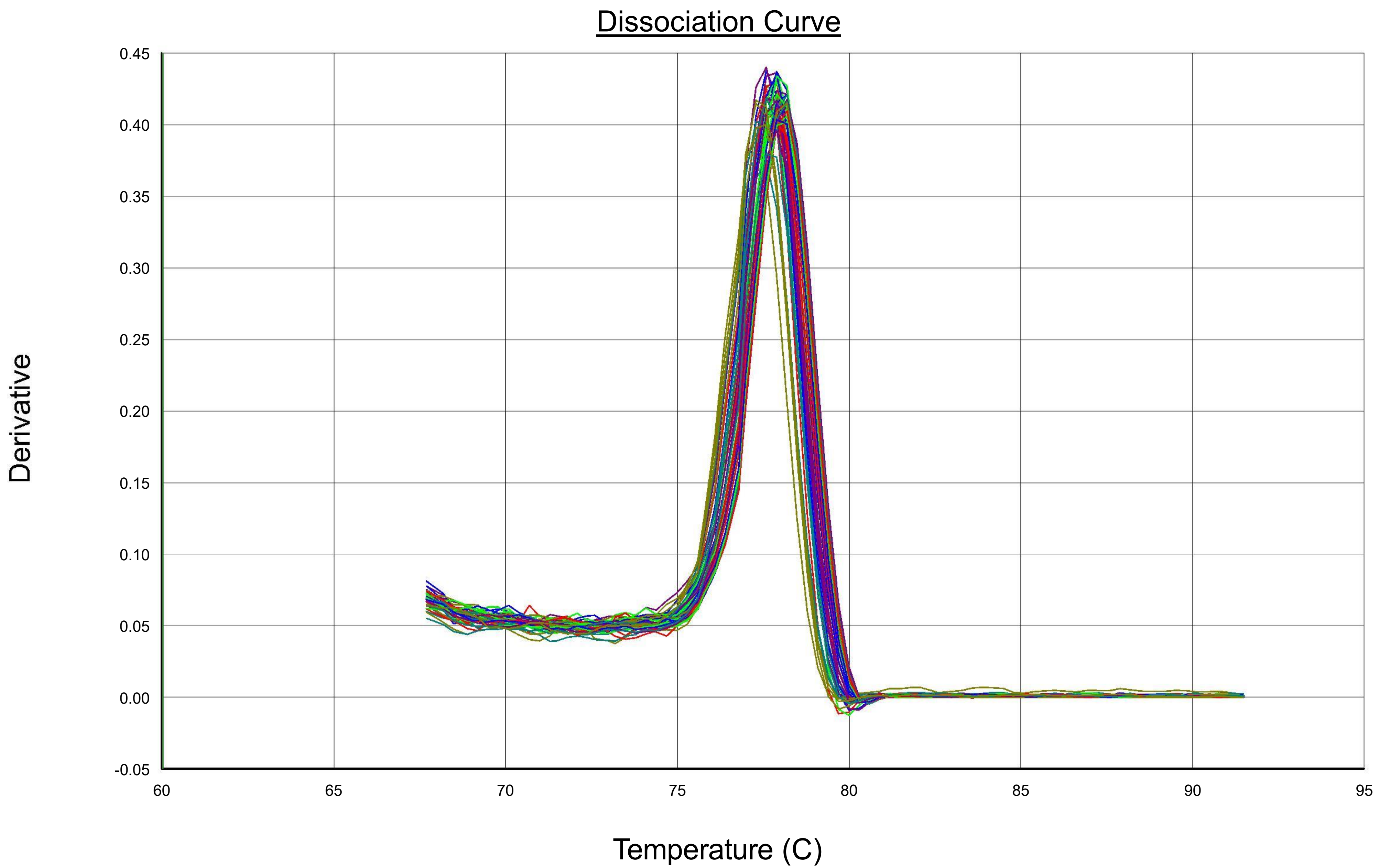

Detector = GUSB_Chicken, Tm = 60.0 °C
Well(s): A1-D12
Document: I_GUSB_plate 2 (Absolute Quantification)

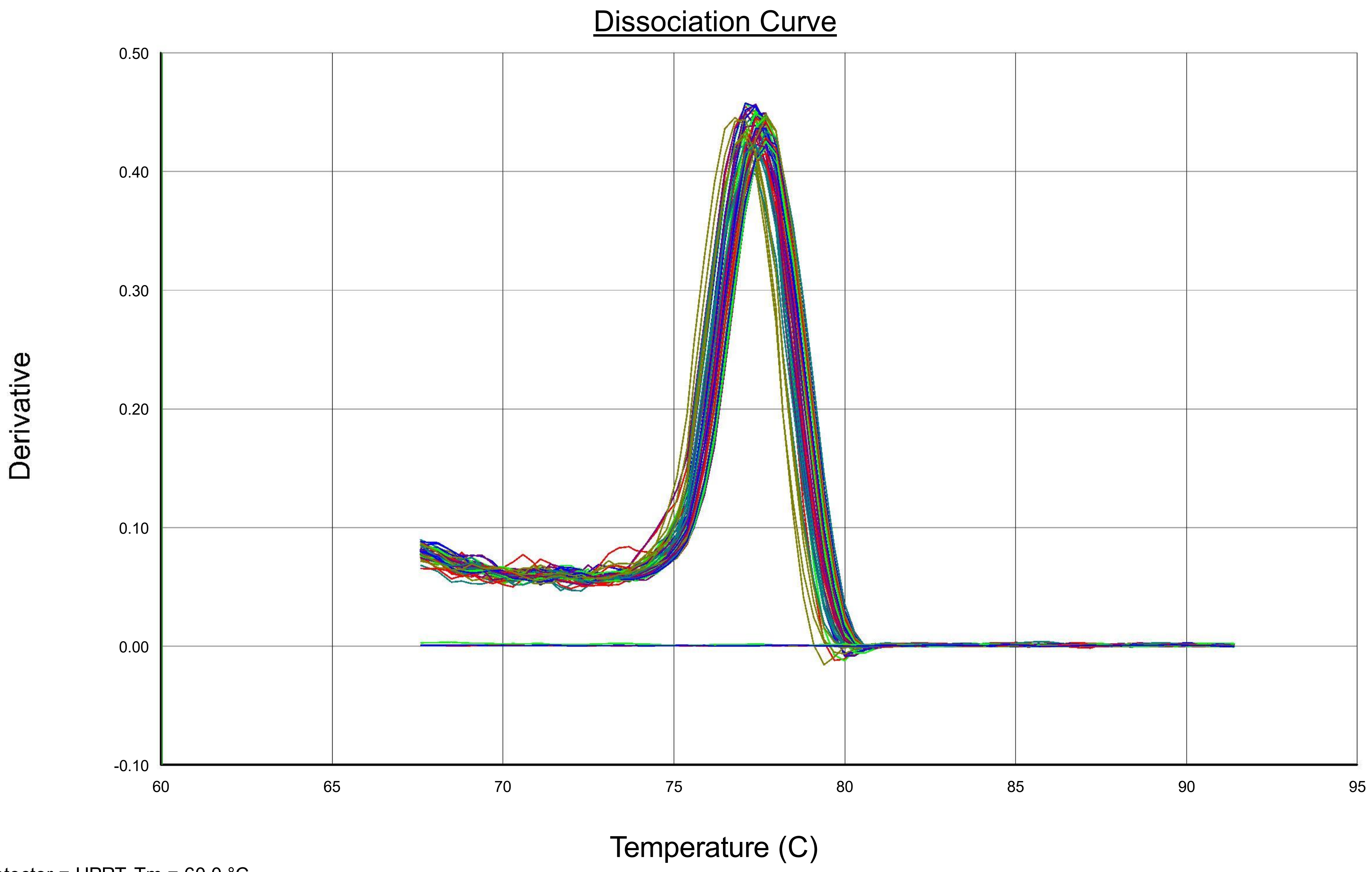

Detector = HPRT, Tm = 60.0 °C
Well(s): A1-D12,H7-H9
Document: I_HPRT_plate 1 (Absolute Quantification)

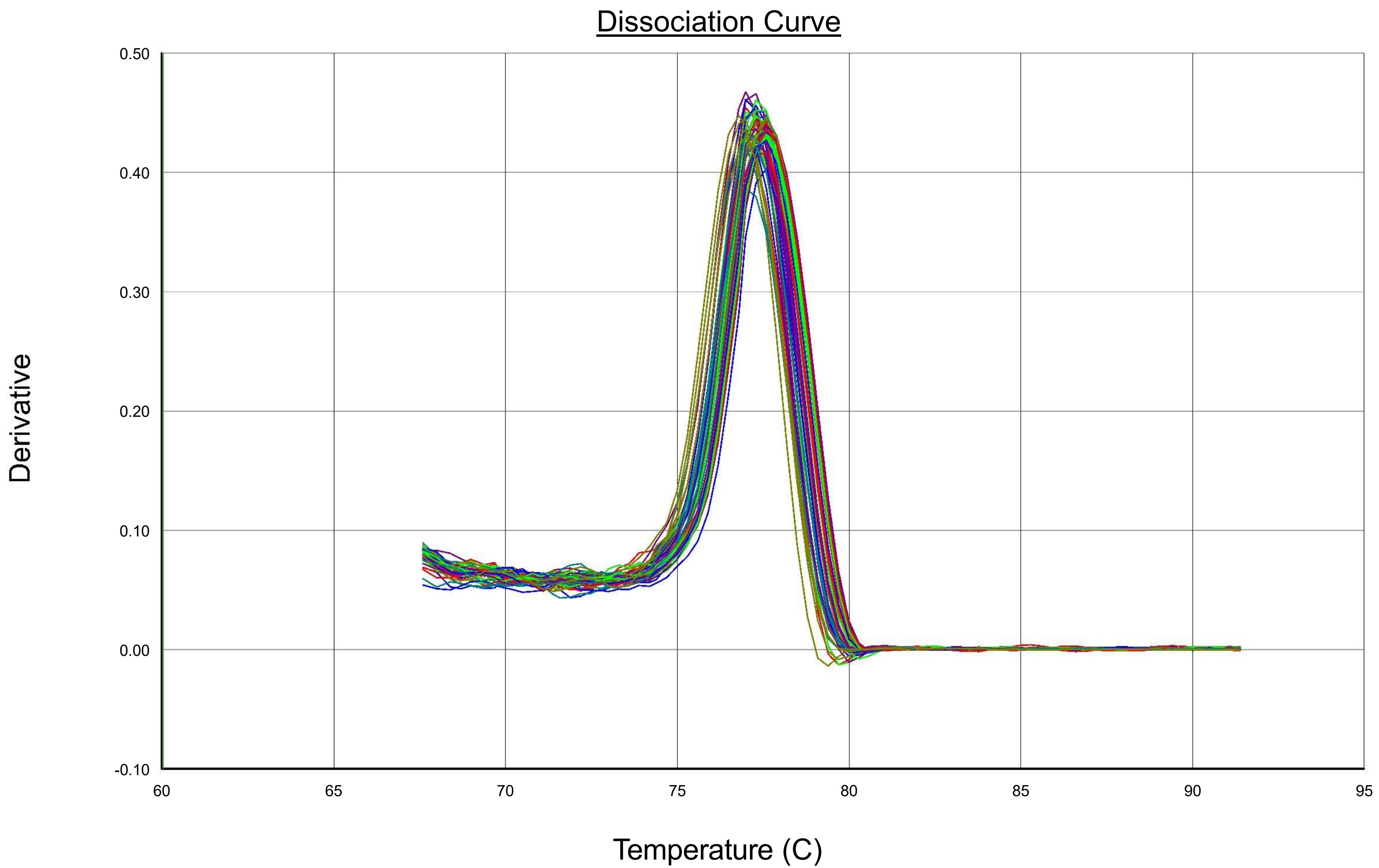

Detector = HPRT, Tm = 60.0 °C
Well(s): A1-D12
Document: I_HPRT_plate 2 (Absolute Quantification)
