# Supplementary material for: Turmeric Powder Counteracts Oxidative Stress and Reduces AFB1 Content in the Liver of Broilers Exposed to the EU Maximum Levels of the Mycotoxin
Source: Toxins (Basel). 2023 Dec 7;15(12):687. doi: 10.3390/toxins15120687 (PMC10747922; doi:10.3390/toxins15120687)

Figure S2. HPLC-FLD chromatograms of AF obtained using the analytical conditions detailed in the par. 5.6.3. The red chromatogram represents a standard calibrant of AF, the green and blue chromatograms are those of liver samples belonging to the AFB1 group and AFB1 plus TP group, respectively.

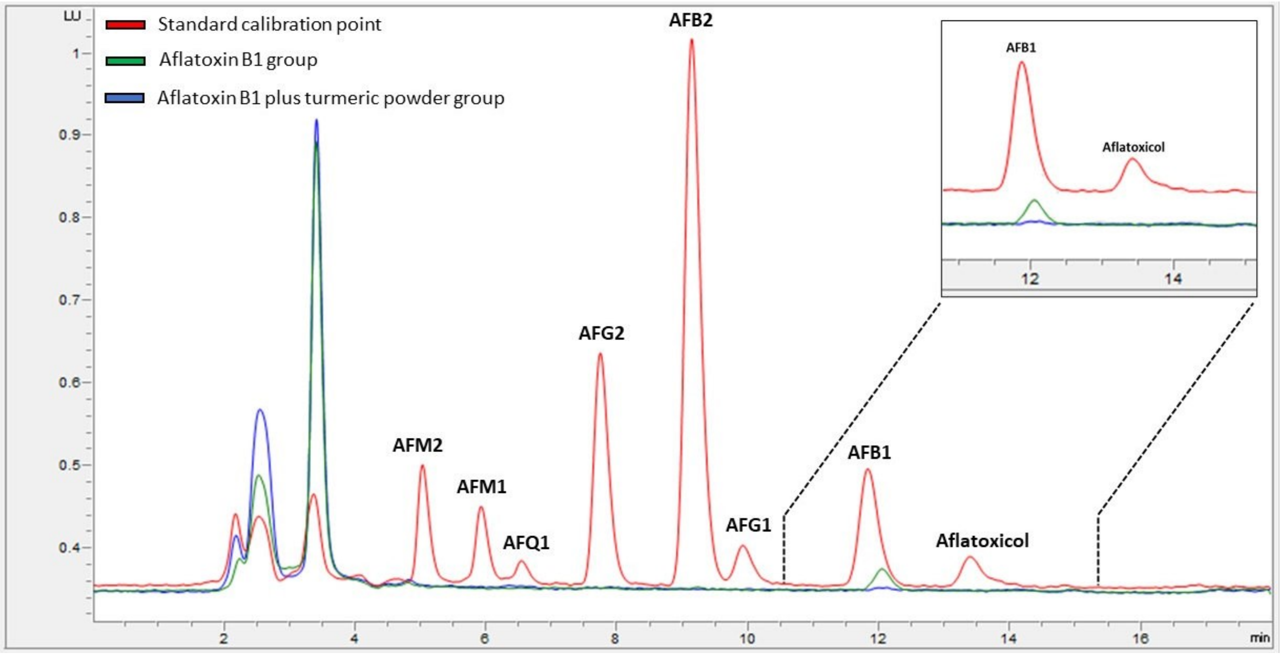

Supplement: Supplementary file 1 [file toxins-15-00687-s001.zip › toxins-2731337-supplementary Figure S2.pdf]
