# Supplementary material for: Turmeric Powder Counteracts Oxidative Stress and Reduces AFB1 Content in the Liver of Broilers Exposed to the EU Maximum Levels of the Mycotoxin
Source: Toxins (Basel). 2023 Dec 7;15(12):687. doi: 10.3390/toxins15120687 (PMC10747922; doi:10.3390/toxins15120687)
Supplement: Supplementary file 1 [file toxins-15-00687-s001.zip › toxins-2731337-supplementary Tables.pdf]

Table S1. Primers for Quantitative Real-Time PCR (qRT-PCR).

Gene	Accession no.	Sequence	Annealing temperature (°C)	Amplicon size (bp)
CYP1A1	NM_205147.1	FW: GATGTCCGCGTCCAACCC<br>REV: GCGGTTGTCACGGTGTCAA	65	86
CYP1A2	NM_205146.2	FW: CGCAGATCCCAAACGAGAAG<br>REV: GCGGTTGTCACGGTGTCAA	62	76
CYP2A6	KX687985	FW: CCCTCTCCTAAACAGATGCG<br>REV: TTGCTGTCTCCCATCCTGC	64	149
CYP2H1	NM_001001616.1	FW: TCCTTCCCCTTAATGTTCCACA<br>REV: GGGAGACAGCAAAGGGAATATC	62	98
CYP3A4	NM_001329508.2	FW: TGGTAGTCATGATCCCAGCC<br>REV: GGGGTCAATGTTCTCTCCGT	66	113
EPHX1	XM_419386.6	FW: TCCTCAATGCGTTTCTACAAAGA<br>REV: TCATTAGGAAAGGAGGCAATGC	63	107
EPHX2	NM_001033645.1	FW: CAAGGGCATGGAGGAGTGG<br>REV: GCCTCTCCATTTGTGTCCAA	66	80
GSTA1	NM_001001777.1	FW: TTTTAGCGGTGGAAGAGTCG<br>REV: GGGGATATTGCTTGTTCTTGCT	62	86
GSTA2	NM_001001776.1	FW: AGCAGCCGATGTGAAAGAAAA<br>REV: GCCAACAAGATAATCCTGACCA	62	115
GSTA4	XM_015284816.2	FW: AGAGAGCCCTGATCGACATG<br>REV: CTCTCTGTTGCCTTCTCTGC	62	130
GSTM2	NM_205090.1	FW: CAACCTGAGCCAATTCCTGC<br>REV: GCGCCGTGTACCAGAAAAT	66	104
Nrf2	NM_205117	FW: AATCAAACTCAGCCACCCAG<br>REV: CAGCCAGGTTGTCGTTTTCA	64	142
CAT	NM_001031215.2	FW: GGCACTGCTGGACAAATACA<br>REV: AAGTGGCTTGCGTGTATGTC	61	71
GPX1	NM_001277853.2	FW: TTCGGGCACCAGGAGAACGC<br>REV: TGGTGAAGTTGGGTTTGAAGC	66	91
SOD1	NM_205064.1	FW: GGGAGGAGTGGCAGAAGTAG<br>REV: CCCTCTACCCAGGTCATCAC	63	115
SOD2	NM_204211.1	FW: GGAGCAGGGACGTCTACAAA<br>REV: CCCAGCAATGGAATGAGACC	62	81
ABCB1	NM_204894.1	FW: ACAACAGTCGGGAGGTGTC<br>REV: GCTGTGTTCCCTTGTCTCCT	62	123
ABCC2	XM_015288821.2	FW: TCCTTGTTCTTTGTCACCACA<br>REV: AGTAGGCAGACACGCGATAA	64	122
ABCG2	NM_001328490.1	FW: TCCTTGTTCTTTGTCACCACA<br>REV: AGTAGGCAGACACGCGATAA	62	124
PGK2	NM_204985.2	FW: CTGCTGGCTTCCTGATGAA<br>REV: TCCTGAACTTTAGCTCCTCCA	62	103
RPS7	XM_001234708.4	FW: GCCCAAGCCAACGAGAAAA<br>REV: TTTACGCGGATTCTCTTGCC	65	138
GUSB	NM_001039316.2	FW: TGATTGGGGAACTCATCTGGA<br>REV: CGTTGGCGGGTAAATATTCCT	62	97
HPRT	NM_204848.1	FW: ACGTTGCTGTCTCTACTTAAGC<br>REV: CCCACACTTCGAGGAGTTCT	61	86

Italicized gene names indicate the internal control genes used as references.

Table S2. Matrix-matched regression curves and related R2 and SSE (%) values

Toxins	Calibration range	n° exp. points	Matrix-matched calibration curve	R^2	SSE (%)
AFB1	0.05-5 ng/mL	5	y=6.12x-0.04	0.999	103
AFB2			y=22.49x+0.09	0.999	98
AFG1			y=2.18x+0.06	0.999	91
AFG2			y=10.45x+0.12	0.999	96
AFM1			y=2.44x+0.03	0.999	96
AFM2			y=2.58x+0.08	0.999	93
AFQ1			y=0.41x-0.02	0.999	90
AFL			y=1.50x+0.01	0.998	107

SSE(%) = signal suppression/enhancement (matrix calibration line slope/standard calibration line slope *100).

Table S3. Limit of detection (LOD, S/N=3) and limit of quantification (LOQ, S/N = 10) calculated for AFB1, AFB2, AFG1, AFG2, AFM1, AFM2, AFQ1 and AFL in liver samples.

	AF concentration (ng/Kg)							
	**AFB1**	**AFB2**	**AFG1**	**AFG2**	**AFM1**	**AFM2**	**AFQ1**	**AFL**
**LOD**	8	2	17	5	17	13	24	30
**LOQ**	25	5	80	16	57	42	80	99

Table S4. Recoveries of AFB1, AFB2, AFG1, AFG2, AFM1, AFM2, AFQ1 and AFL in liver samples spiked at 2 different AFBs levels (0.1 and 1 ng/g).

Spiking level (ng/Kg)	Recovery, % (RSD,%, *n*=3)							
	**AFB1**	**AFB2**	**AFG1**	**AFG2**	**AFM1**	**AFM2**	**AFQ1**	**AFL**
**1000**	87 (7)	92 (7)	99 (9)	85 (9)	91 (9)	20 (3)	29 (8)	90 (7)
**100**	90 (1)	89 (2)	93 (7)	73 (2)	83 (3)	<LOD	<LOD	93 (7)

Determination of AF was performed using the optimized analytical method and quantification was performed using the standard calibration line. RSD = relative standard deviation.
